# The human proton pump inhibitors inhibit *Mycobacterium tuberculosis* rifampicin efflux, and macrophage-induced rifampicin tolerance

**DOI:** 10.1073/pnas.2215512120

**Published:** 2023-02-10

**Authors:** M. Alexandra Lake, Kristin N Adams, Feilin Nie, Elaine Fowler, Amit K Verma, Silvia Dei, Elisabetta Teodori, David R Sherman, Paul H Edelstein, David R Spring, Mark Troll, Lalita Ramakrishnan

**Affiliations:** a Molecular Immunity Unit, Cambridge Institute of Therapeutic Immunology and Infectious Diseases, Department of Medicine, University of Cambridge, CB2 0AW Cambridge, United Kingdom; b Medical Research Council Laboratory of Molecular Biology, CB2 0QH Cambridge, United Kingdom; c Department of Microbiology, University of Washington, Seattle, United States; d Yusuf Hamied Department of Chemistry, University of Cambridge, Lensfield Road, Cambridge CB2 1EW, UK; e Department of Neuroscience, Psychology, Drug Research and Child Health - Section of Pharmaceutical and Nutraceutical Sciences, University of Florence, via Ugo Schiff 6, 50019 Sesto Fiorentino (FI), Italy; f Perelman School of Medicine, University of Pennsylvania, Philadelphia, PA

## Abstract

Tuberculosis treatment requires months-long combination chemotherapy with multiple drugs, with shorter treatments leading to relapses. A major impediment to shortening treatment is that *Mycobacterium tuberculosis* becomes tolerant to the administered drugs, starting early after infection and within days of infecting macrophages. Multiple lines of evidence suggest that macrophage-induced drug tolerance is mediated by mycobacterial drug efflux pumps. Here, using assays to directly measure drug efflux, we find that *M. tuberculosis* transports the first line antitubercular drug rifampicin through a proton gradient-dependent mechanism. We show that verapamil, a known efflux pump inhibitor, which inhibits macrophage-induced rifampicin tolerance, also inhibits *M. tuberculosis* rifampicin efflux. As with macrophage-induced tolerance, the calcium channel-inhibiting property of verapamil is not required for its inhibition of rifampicin efflux. By testing verapamil analogs, we show that verapamil directly inhibits *M. tuberculosis* drug efflux pumps through its human P-glycoprotein-like inhibitory activity. Screening commonly used drugs with incidental P-glycoprotein inhibitory activity, we find many inhibit rifampicin efflux, including the proton pump inhibitors like omeprazole. Like verapamil, the proton pump inhibitors inhibit macrophage-induced rifampicin tolerance as well as intramacrophage growth, which has also been linked to mycobacterial efflux pump activity. Our assays provide a facile screening platform for *M. tuberculosis* efflux pump inhibitors that inhibit in vivo drug tolerance and growth.

## Introduction

Tuberculosis requires months-long multidrug treatment for reliable cures (1), with shorter regimens leading to increased rates of relapse (1–4). Because relapses typically involve genetically drug-susceptible organisms, they are attributed to recrudescence of residual organisms with phenotypic drug resistance, also known as tolerance (5). Drug-tolerant organisms do not have genetic drug resistance mutations and are killed normally under standard laboratory conditions but poorly within the host (5).

Mtb drug tolerance has long been attributed to metabolically quiescent or dormant populations (6). In recent years, we have uncovered a distinct mechanism for Mtb drug tolerance where bacteria become tolerant to multiple antitubercular drugs upon entering macrophages (7). In contrast to the dormant Mtb tolerance models (6), macrophage-induced tolerance is enriched in actively-growing bacteria (7). Importantly, macrophage-induced tolerance occurs in diverse Mtb clinical isolates from all four common lineages (8).

Two lines of evidence suggest that macrophage-induced drug tolerance is mediated by Mtb drug efflux pumps. In the virulent laboratory strain, CDC1551, genetic knockdown of the major facilitator superfamily (MFS) efflux pump TAP (Rv1258c), which is transcriptionally induced when Mtb infects macrophages (9), causes loss of macrophage-induced rifampicin tolerance. Consistent with this finding, among Mtb clinical isolates, only the lineage 2 Beijing strains with a loss-of-function mutation in Rv1258c fail to develop macrophage-induced rifampicin tolerance (8). In the second line of evidence, macrophage-induced drug tolerance is inhibited by multiple structurally distinct drugs that are known to inhibit bacterial efflux pumps (7, 10, 11). Rv1258c also constitutes a virulence determinant, promoting Mtb intramacrophage growth in the absence of antibiotics and likewise, the efflux blocking drugs inhibit intramacrophage Mtb growth in the absence of antibiotics (7, 8, 10). Verapamil, the best studied of these drugs, was shown to decrease relapse rates in mice infected with drug-sensitive TB and given shortened courses of standard antitubercular treatment (12). Furthermore, verapamil significantly reduced bacterial loads in mice infected with a multidrug resistant Mtb strain (i.e., rifampicin and isoniazid resistant) treated with the standard first line rifampicin-containing regimen (13).

Here, we develop and validate assays to directly measure Mtb rifampicin efflux and link it to macrophage-induced rifampicin tolerance and intramacrophage growth in the absence of antibiotics. We leverage these assays to identify the PPIs as a new class of approved drugs that inhibit Mtb macrophage-induced tolerance. Finally, our findings support a large body of work finding that verapamil acts directly to inhibit bacterial efflux pumps.

## Results

### Verapamil inhibits rifampicin efflux from axenically-grown Mtb

**Figure 1:**
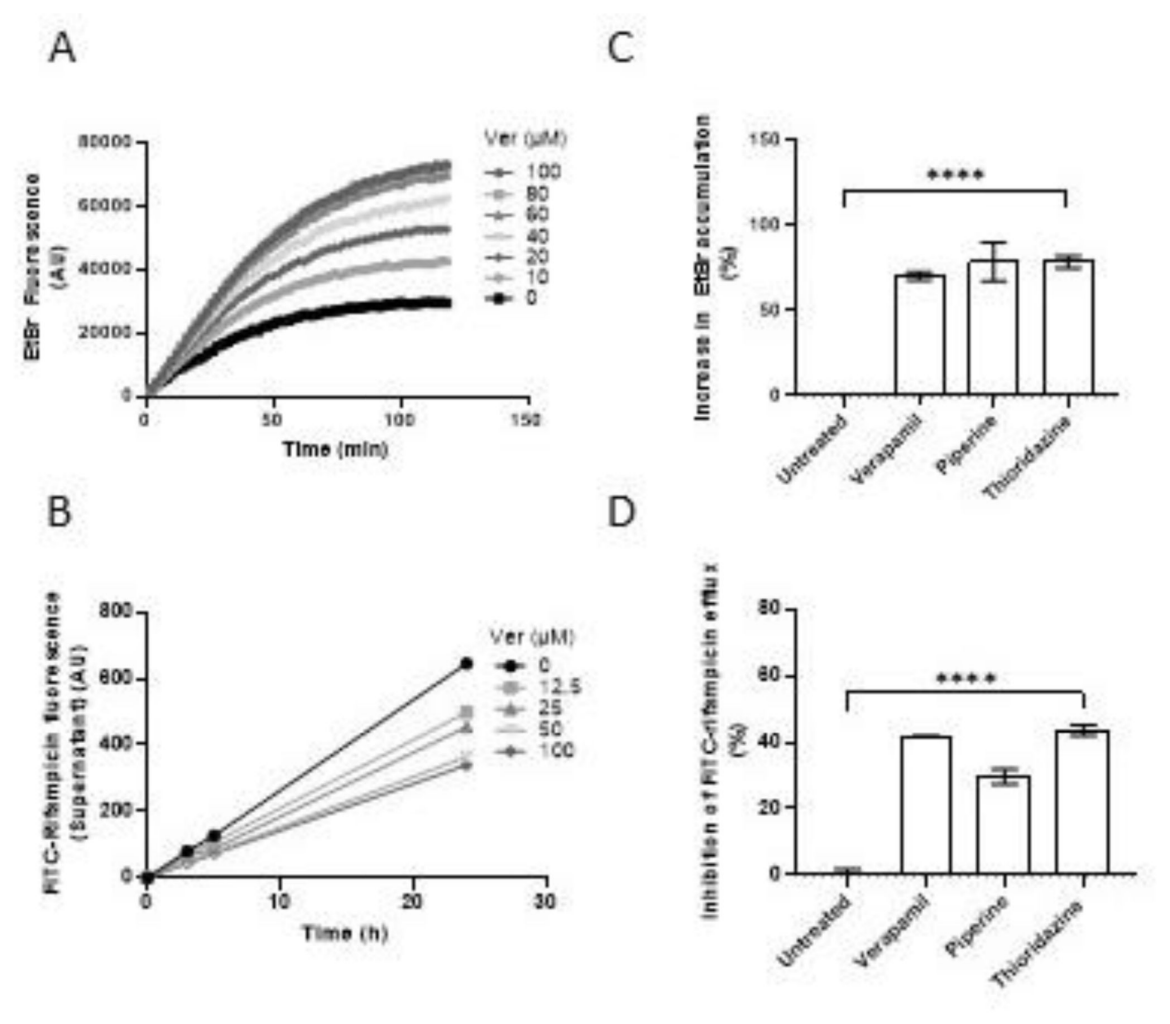
Efflux of FITC-rifampicin and ethidium bromide (EtBr) in broth from Mtb recapitulates in vivo inhibitor activity. (**A**) Mtb mc^2^6206 intracellular ethidium bromide (EtBr) fluorescence over time in the presence of verapamil (Ver) 0-100 μM. Values represent mean of three technical replicates. (**B**) Efflux of FITC-rifampicin into supernatant over time from cells of Mtb mc^2^6206 treated with Ver 0-100 μM. (**C**) Percent increase in intracellular EtBr accumulation in Mtb mc^2^6206 due to Ver 160μM, piperine 350μM or thioridazine 5.4 μM, normalised to mean untreated value. (**D**) Percent inhibition of FITC-rifampicin efflux from cells of Mtb mc^2^6206 due to Ver 160μM, piperine 350μM or thioridazine 5.4 μM, normalised to mean untreated value. (**B,C**,**D**) Values represent mean of three technical replicates ± SEM. Statistical analysis by one-way ANOVA with Dunnett’s multiple comparisons test. **** = *P* <0.0005.

We used two assays to assess rifampicin efflux from axenically grown Mtb, using the H37Rv mc^2^6206 strain, a genetically-defined leucine and pantothenic acid auxotroph derived from the commonly used virulent strain H37Rv suitable for biosafety level 2 facilities (14–18). In the first assay, we measured ethidium bromide accumulation as a surrogate for rifampicin efflux. The ethidium bromide (EtBr) accumulation assay uses the artificial efflux pump substrate EtBr, the fluorescence of which increases 20-fold when intercalated with DNA (19). Thus, when its efflux is blocked, its accumulation inside bacterial cells, including mycobacteria, renders them more fluorescent (SI Appendix, Fig. 1A) (20–23). Fluorescence can be measured continuously in a plate reader, and typically rises from baseline over 90-180 minutes until a stable steady state is reached between influx and efflux, and fluorescence levels cease to change. Using this assay, we found the expected time-dependent increase in bacterial fluorescence, indicating progressive EtBr accumulation. As shown before (22, 24–26), verapamil increased EtBr accumulation in a concentration-dependent manner at concentrations as low as 5% of its minimum inhibitory concentration (MIC) for H37Rv mc^2^6206 (Fig. 1A; SI Appendix, Table S1). These concentrations were also within the range of those required to inhibit macrophage-induced rifampicin tolerance (10).

To specifically assay rifampicin efflux, we used rifampicin conjugated to the fluorophore fluorescein isothiocyanate (FITC). We chose FITC for its relatively small size and used a long, flexible carbon linker to minimize its steric hindrance. (SI Appendix, Fig. S2A and supplemental methods). The FITC-rifampicin conjugate retained some activity against Mtb (MIC 16-fold the rifampicin MIC) (SI Appendix, Table S1), comparable to the 9-fold MIC increase reported for a different FITC-rifampicin (27). To measure rifampicin efflux, we loaded bacteria with FITC-rifampicin at 37°C for 30 minutes, washed at 4°C to remove conjugate adhering to bacterial surfaces, then measured supernatant fluorescence over time at 37°C. In contrast to the EtBr accumulation assay, measurement was not continuous but by intermittent sampling to separate supernatant from bacteria for analysis (SI Appendix, Fig. S1B). Supernatant fluorescence increased over the assay period, indicating time-dependent efflux (SI Appendix, Fig S2B). To ensure that our assay was measuring active rifampicin transport rather than passive diffusion, we performed the assay at 4°C, which was reported to inhibit active transport but not passive diffusion (28). No significant rifampicin efflux was observed from Mtb maintained at 4°C, confirming that our assay is a measure of active transport (SI Appendix, Fig S2B). To confirm that FITC-rifampicin is transported by the same route as rifampicin, we set up a competition assay by loading cells with 2μM FITC-rifampicin and 4μM unlabeled rifampicin in the assay. The unlabeled rifampicin significantly decreased FITC-rifampicin efflux at 90 minutes, suggesting that FITC-rifampicin is transported by the same efflux pumps as rifampicin (SI Appendix, Fig. S2C) While EtBr accumulation reaches a final steady state level within 1-2 hours, FITC-rifampicin efflux continued for at least 24 hours. For ease of comparison between assays, we adopted a final 24 hour end point in the FITC-rifampicin assay.

Verapamil reduced FITC-rifampicin efflux in a concentration-dependent manner down to 5μM (Fig. 1B and SI Appendix, Fig S3A); these concentrations were similar to those inhibiting EtBr efflux (Fig. 1A). Verapamil had no effect on efflux of unconjugated FITC, showing that its reduction of FITC-rifampicin efflux is specifically due to its effect on rifampicin efflux (SI Appendix, Fig S3B). Next, we tested two other drugs, piperine and thioridazine, which inhibit bacterial efflux pumps in vitro and macrophage-induced rifampicin tolerance (10, 11, 29). As shown before, both drugs inhibited EtBr efflux (26, 30) (Fig. 1C). They also inhibited rifampicin efflux (Fig. 1D). All three drugs were used at concentrations that do not inhibit Mtb growth over 48 hours (10). Nevertheless, to ensure that any observed reductions in FITC-rifampicin efflux were not due to microbicidal effects, we performed the assay on heat-killed Mtb. In contrast to the effects of the compounds on live bacteria, loss of Mtb viability increased FITC-rifampicin release into the medium, with the addition of verapamil (25μM) having no further effect (SI Appendix, Fig. S2D.) This result is consistent with nonviable Mtb having decreased cell wall integrity, and confirms the specificity of the assay. Thus, three structurally distinct compounds inhibit both rifampicin efflux from axenically-grown Mtb as well as efflux-pump mediated rifampicin tolerance that develops when Mtb becomes macrophage resident. This finding suggested that pumps with overlapping substrates and inhibitors mediate in vivo and in vitro efflux.

### Rifampicin efflux is mediated principally by proton gradient-dependent transporters

**Figure 2:**
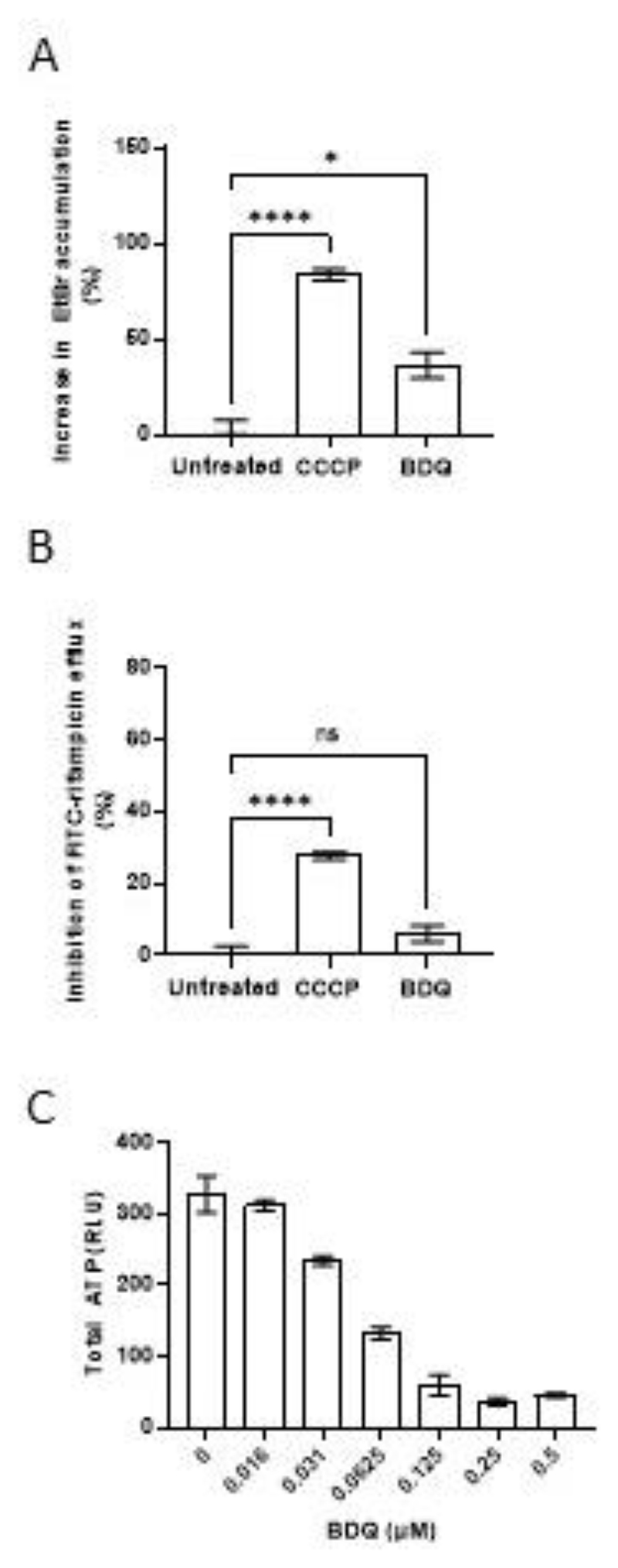
Rifampicin efflux is mediated principally by proton gradient-dependent transporters (**A**) Percent increase in Mtb mc^2^6206 intracellular EtBr accumulation due to CCCP 6.25μM or bedaquiline (BDQ) 0.0625μM (25% of MIC).(**B**) Percent inhibition of FITC-rifampicin efflux from cells of Mtb mc^2^6206 due to CCCP 6.25μM or BDQ 0.0625μM. (**C**) Total Mtb mc^2^6206 ATP after 24 hours treatment with BDQ. RLU, relative luminescence units. Values represent mean of three technical replicates ± SEM. **** = *P* <0.0005. Representative of at least two experiments at similar concentrations. Statistical analysis by one-way ANOVA with Dunnett’s multiple comparisons test.

Most bacterial, including mycobacterial, drug efflux pumps belong to one of four major classes: the adenosine triphosphate (ATP)-binding cassette (ABC) transporter class powered by ATP hydrolysis with the remaining three, the MFS, resistance-nodulation-division (RND), and small multidrug resistance (SMR) group pumps being ATP-independent and exclusively dependent on the membrane proton gradient for transport (11). Many mycobacterial efflux pumps that are macrophage-induced, or are involved in virulence, belong to the ABC family or MFS families, including the MFS transporter TAP/Rv1258c, which mediates macrophage-induced rifampicin tolerance (7, 10). We confirmed that Rv1258c is a major contributor to macrophage-induced rifampicin tolerance – not only was tolerance lost in Rv1258c mutant Mtb but the addition of verapamil had no additional effect (SI Appendix, Fig S4A). However, modulation of Rv1258c expression in axenic culture, where Rv1258c is present at only basal levels (9), has minimal effects on rifampicin susceptibility (31–34). It was therefore unlikely to contribute to the rifampicin efflux observed in these assays. We confirmed this by testing a Rv1258c mutant in the Mtb H37Ra strain, which, like Mtb H37Rv mc^2^6206, is suitable for containment level 2 use. The TAP/Rv1258c mutant exhibited neither altered EtBr accumulation nor FITC-rifampicin efflux, and retained verapamil sensitivity similar to the isogenic H37Ra parent strain, suggesting that low Rv1258c expression precluded its contributing to rifampicin efflux (Figure S4B and C). We asked if other proton gradient-dependent transporters were involved by testing the effect of the protonophore carbonyl cyanide m-chlorophenyl hydrazone (CCCP) (35) at 25% of its MIC for H37Rv mc^2^6206 (SI Appendix, Table 1). CCCP strongly inhibited both rifampicin and EtBr efflux (Fig 2A and B). In contrast the ATP synthase inhibitor bedaquiline (35) at 25% of its MIC for H37Rv mc^2^6206 (SI Appendix, Table 1) had no significant effect on rifampicin efflux and only a modest effect on EtBr accumulation (Fig. 2A and B). We confirmed that bedaquiline showed the expected inhibition of ATP synthesis: it depleted 59.5 ± 2.6% (mean ± SEM) of total bacterial ATP at the concentration used in the efflux assays (Fig. 2C). This finding could explain the shared inhibitory activity of the three drugs on efflux in vitro and macrophage tolerance and lend further support for screening for inhibition of EtBr/rifampicin efflux in vitro as an initial step to identify inhibitors of drug tolerance in vivo.

### Verapamil’s inhibition of Mtb efflux pumps is linked to its human P-glycoprotein inhibitory activity

Verapamil has two distinct activities that are dissociable from each other. It inhibits calcium channels, which is the basis of its clinical use as a cardiovascular drug, and it inhibits a major human promiscuous multi-drug ABC transporter, P-glycoprotein (PGP)(36, 37). We have shown that verapamil inhibits macrophage-induced rifampicin tolerance independent of its calcium channel blocking activity; its R-enantiomer and its major metabolite norverapamil, both with reduced cardiac activity thought to be due to their lower inhibitory activity of calcium channel blocker in relation to S-verapamil (38, 39), inhibit rifampicin tolerance with similar efficacy to racemic verapamil (10).

**Figure 3:**
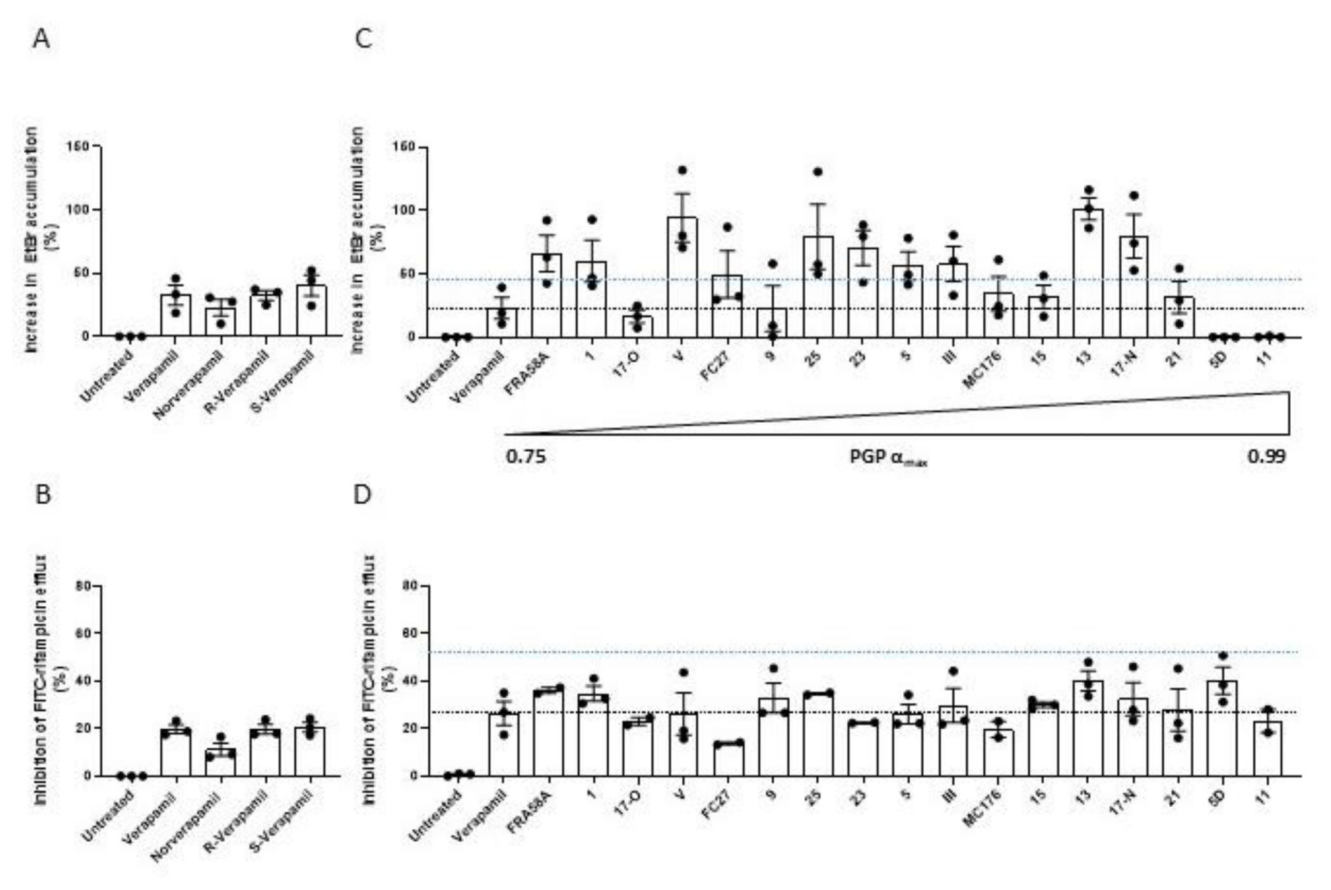
Verapamil analogues targeting P-glycoprotein increase accumulation of EtBr and inhibit the efflux of FITC-rifampicin in Mtb. (**A,C**) Percent increase in intracellular EtBr accumulation in Mtb mc^2^6206 due to 25μM drug. (**B,D**) Percent inhibition of FITC-rifampicin efflux from cells of Mtb mc^2^6206 due to 25μM drug. (**C,D**) Drugs ordered left to right by increasing efficacy of PGP inhibition (α_max_). α_max_ is the maximum increase in the nuclear concentration of the PGP substrate pirarubicin in eukaryotic pirarubicin-resistant cells that can be obtained with a given compound, where α varies between 0 (no inhibitor present) and 1 (when the amount of pirarubicin in resistant cells is the same as in sensitive cells). Blacked dashed line indicates mean value for verapamil; blue dashed line indicates 2-fold verapamil activity. (**C**,**D**) Gaussian distribution of data was confirmed by assessment of QQ plot then outliers within each experiment were detected using Grubbs’ test (α=0.05) and removed and remaining values normalised to the mean untreated value. (**A-D**) Each symbol represents the mean normalised value for that drug for one individual experiment. (**A**,**B**,**C**) Combined data of three experiments. (**D**) Combined data of two (compounds 11, 17-Old, 23, 25, FC27, MC176,FRA58A) or three experiments.

Accordingly, R-verapamil inhibited both EtBr and rifampicin efflux to the same extent as racemic verapamil and its cardiac-active S-enantiomer (Fig. 3A and B), suggesting a non-calcium channel mechanism such as PGP inhibition. Norverapamil also inhibited both efflux activities albeit less than the other compounds (Fig. 3A and B), However, at higher norverapamil concentrations, which inhibit macrophage-induced tolerance (10), rifampicin efflux was inhibited to a similar degree as verapamil (SI Appendix, Fig. S5). This finding was consistent with the inhibition of rifampicin efflux by verapamil being independent of its calcium channel activity, like its inhibition of rifampicin tolerance, and suggested that it was instead linked to its PGP-inhibiting activity.

To probe this, we screened a panel of seventeen verapamil analogues which specifically target PGP with negligible cardiac effects, showing that they had minimal calcium channel inhibitory activity (SI Appendix, Table S2) (40–46). These compounds were enriched for inhibitors of both EtBr and rifampicin efflux. Fifteen inhibited EtBr efflux, ten with two-fold or greater potency than verapamil (Fig. 3C and SI Appendix, Table S3). All inhibited rifampicin efflux, many with similar or greater (albeit < two-fold) efficacy than verapamil (Fig. 3D and SI Appendix, Table S4). There was no correlation between the strengths of PGP and mycobacterial efflux pump inhibition, which is not surprising given that PGP and bacterial transporters will have structural differences (Fig. 3C and D, SI Appendix Fig. S6A and B and Table S3 and S4). Importantly, there was also not a good correlation for strength of inhibition of EtBr and FITC-rifampicin efflux. Two of the compounds, 5D and 11, had no activity against EtBr efflux and good activity against rifampicin efflux (Fig. 3C and D and SI Appendix Fig. S6C). Thus, distinct bacterial transporters with mostly but not completely overlapping substrate specificities may transport these two substances. Overall, these findings suggested that screening PGP inhibitors for Mtb efflux inhibition can identify new tolerance-inhibiting drugs.

### The proton pump inhibitor (PPI) class of drugs are potent inhibitors of rifampicin efflux

**Figure 4:**
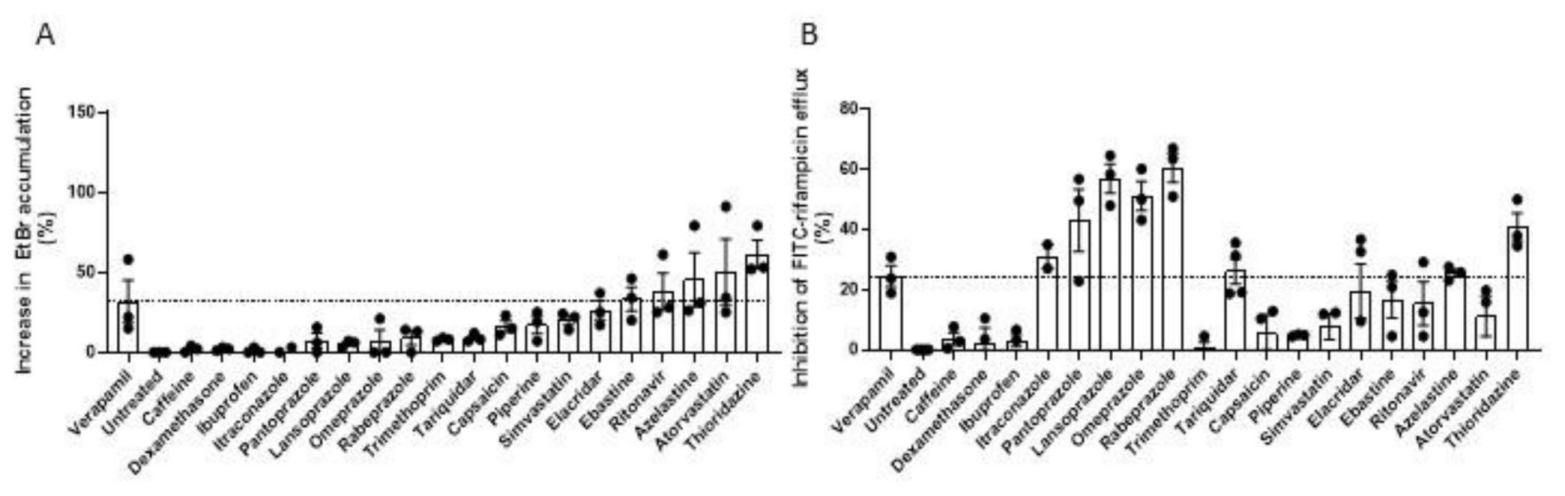
Omeprazole, lansoprazole, rabeprazole, and pantoprazole are potent inhibitors of rifampicin but not EtBr efflux. (**A**) Percent increase in intracellular EtBr accumulation in Mtb mc^2^6206 due to 25μM drug. (**B**) Percent inhibition of FITC-rifampicin efflux from cells of Mtb mc^2^6206 due to 25μM drug. (**A,B)** Dashed line indicates mean value for verapamil. Gaussian distribution of data was confirmed by assessment of QQ plot then outliers within each experiment were detected using Grubbs’ test (α=0.05) and removed, then remaining values normalised to the mean untreated value. Each symbol represents the mean normalised value for that drug for one individual experiment. Column height and error bars represent mean ± SEM of all mean values shown for that drug. (**A**) Combined data of three experiments. (**B**) Combined data of three experiments, except for itraconazole, which was tested twice.

PGP inhibition is a well-documented ‘off-target’ effect of many approved drugs (47). We screened a panel of drugs with incidental PGP inhibitory activity for inhibition of Mtb EtBr and rifampicin efflux inhibition, prioritizing inexpensive, widely available drugs using DrugBank (48). This panel was also enriched for inhibition of EtBr and FITC-rifampicin efflux (Fig. 4A and B, and SI Appendix, Table S3 and Table S4). As with the verapamil analogs, there was not a correlation in the strength of the compounds for inhibition of efflux of the two substrates (SI Appendix Fig. S4D). However, there was some concordance in whether they had efflux activity. Five and ten of the 19 drugs respectively inhibited EtBr and rifampicin efflux, with three significantly inhibiting both (Fig. 4A and B). One drug, atorvastatin, inhibited EtBr well but was a poor inhibitor of rifampicin efflux (Fig. 4A and B). The most striking difference was that the four proton pump inhibitors (PPIs), omeprazole, lansoprazole, pantoprazole and rabeprazole, showed negligible inhibition of EtBr efflux and were the strongest inhibitors of rifampicin efflux (Fig. 4A and B) at the screening concentration of 25μM. Indeed, all four PPIs inhibited rifampicin efflux even at 2.5μM, the lowest concentration tested (SI Appendix, Fig. S7A). This concentration was far (40-160-fold) below the MIC of these drugs (SI Appendix, Table S1). However, we further confirmed that their effects on rifampicin efflux were specific rather than due to altered bacterial viability affecting cell wall permeability. The fluorescent nuclear dye propidium iodide has increased penetration into killed Mtb due to decreased cell wall integrity, for which it is a sensitive assay (SI Appendix, Fig. S7B). Using rabeprazole as a representative of the PPI class, we found that it did not cause any increase in propidium iodide uptake after 24 hours exposure of FITC-rifampicin-loaded cells up to a concentration of 100μM, its MIC for Mtb (SI Appendix, Fig. S7B and Table S1). Together, these findings reinforce our findings that Mtb efflux pump inhibitors are enriched among drugs with incidental PGP-inhibiting activity.

### The PPIs inhibit Mtb macrophage-induced rifampicin tolerance and intramacrophage growth in the absence of antibiotics

**Figure 5:**
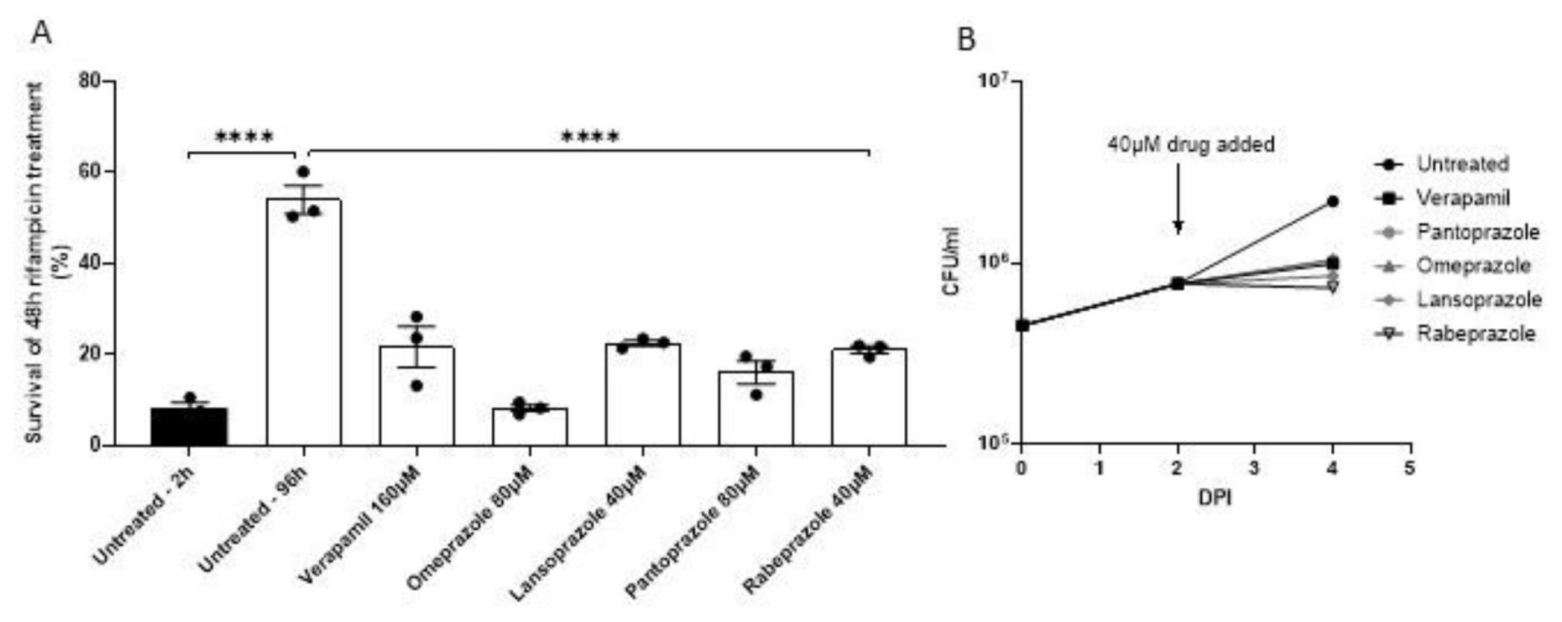
The proton pump inhibitors inhibit intramacrophage growth and rifampicin tolerance of Mtb. (**A**) THP-1 macrophages were infected with Mtb H37Rv and lysed 2 hours (black bars) or 96 hours (white bars) after infection. The released bacteria were treated for an additional 48 hours with 1 μg/mL rifampicin with or without inhibitor before enumeration of CFU. Statistical analysis by ordinary one-way ANOVA with Dunnett’s multiple comparisons test. Representative of 2 independent experiments. (**B**) Growth of Mtb H37Rv in THP-1 macrophages infected at MOI 1. 40μM drug or vehicle was added at 2 days post infection (DPI) and growth allowed to continue a further 48h. Macrophages were lysed in H_2_O and the lysate plated in dilution for colony-forming units (CFU). Results representative of 2 or more independent experiments, except lansoprazole 40μM, which was tested once. **A,B** Error bars represent SEM. **** *P* <0.0005.

Testing verapamil in a direct efflux assay led us to identify PGP inhibiting drugs as potent inhibitors of rifampicin efflux in axenically-grown Mtb. So far, we had validated our in vitro efflux assays by showing that all three drugs that inhibit rifampicin drug tolerance in vivo also inhibited in vitro efflux. We now wanted to go back full circle to determine if the drugs identified in the in vitro assays inhibit intracellular drug tolerance. We selected for testing the four proton pump inhibitors, omeprazole, pantoprazole, lansoprazole and rabeprazole for two reasons: 1) they are among the most widely-used drugs, inexpensive with an excellent tolerability and safety profile (49–51); and 2) their selective inhibition of rifampicin but not EtBr efflux would allow us to test which of the assays was more relevant in vivo. Because Mtb mc^2^6206 is attenuated for growth in THP-1 macrophages (52), we used the parent strain Mtb H37Rv which exhibits macrophage-induced rifampicin tolerance similar to clinical isolates (7, 8, 10). Macrophage-induced Mtb tolerance to antimicrobial agents is defined as the occurrence of reduced antimicrobial killing following *M. tuberculosis* macrophage residence (7, 10). Tolerance to rifampicin develops by 96 hours of macrophage residence (7, 10). We infected the THP-1 human macrophage cell line with Mtb H37Rv for 96 hours, then lysed the cells and incubated the lysates for 48 hours with 1μg/ml rifampicin (5-fold its MIC for this strain) with or without the individual PPIs (7, 8, 10)(SI Appendix, Fig. S8). Verapamil was used at 160μM (50% of MIC) as before (10). We adjusted the PPI concentrations to be at 25-50% of the verapamil concentration and 50% or more below their MICs for H37Rv mc^2^6206 (SI Appendix, Table S1). As expected (7, 8, 10), Mtb developed rifampicin tolerance after 96 hours of macrophage growth, which was inhibited by verapamil (Fig. 5A). All four PPIs inhibited the tolerance similarly, despite being used at 25-50% of the verapamil concentration and at sub-MIC concentrations (Fig. 5A). Verapamil, as well as the other two drugs – thioridazine and piperine – also inhibits Mtb intramacrophage growth in the absence of antimicrobials (7, 8, 10), likely by disrupting Mtb-induced efflux from the rifampicin-transporters that also transport host molecules that are toxic to the bacteria (7, 8, 10). We found that all four PPIs inhibited Mtb intramacrophage growth similarly to verapamil (Fig. 5B). Thus, our in vitro rifampicin efflux assay allowed us to identify the PPIs as inhibitors of Mtb macrophage-induced rifampicin tolerance and intramacrophage growth with similar or greater potency than verapamil.

## Discussion

Our whole-cell Mtb efflux assays strengthen the link between rifampicin drug efflux pumps and macrophage-induced drug tolerance, confirm that verapamil inhibits efflux and drug tolerance by direct inhibition of Mtb efflux pumps and demonstrate the success of a relatively facile platform for screening for inhibitors of in vivo tolerance. We decided to study efflux in axenically-grown Mtb because it was not technically feasible to do so from macrophage-resident bacteria - the relatively small numbers of bacteria that can be obtained from macrophages would be insufficient for the assays. We reasoned that axenically-grown Mtb would be relevant to in vivo conditions even if the exact pumps mediating efflux in vitro and in vivo were different; for instance, TAP/Rv1258c which mediates macrophage-induced tolerance, is expressed at relatively low levels in axenically-grown Mtb and is transcriptionally induced in macrophages (9, 53). We were optimistic because verapamil, which inhibits macrophage-induced rifampicin tolerance, is a well-known bacterial efflux pump inhibitor (11) and inhibits EtBr efflux from axenically-grown Mtb (2–5). Furthermore, verapamil inhibits macrophage-induced tolerance to rifampicin, isoniazid and bedaquiline, all likely mediated by distinct efflux pumps (10).Together these findings suggested that verapamil inhibits different pumps with overlapping and even distinct substrate specificities. We tested this by showing that verapamil inhibits not just EtBr efflux but also rifampicin efflux from axenically-grown Mtb. The latter finding is important as previous studies yielded conflicting results. A study using LC/MS to measure rifampicin levels did not detect an increase in its accumulation in Mtb treated with verapamil (55) whereas a study using radiolabeled rifampicin accumulation over one and two hours found increased intracellular accumulation of ^3^H- rifampicin in the presence of verapamil (56). Further strengthening the link between drug efflux in vitro and in vivo efflux (reflected by macrophage-induced drug tolerance), we find that verapamil inhibits through its PGP inhibitory rather than calcium channel inhibitory activity. Importantly, PGP inhibiting drugs discovered through the in vitro efflux assays inhibit efflux pump-linked mycobacterial growth and rifampicin tolerance in macrophages.

Our finding that many drugs with PGP inhibitory activity inhibit mycobacterial drug efflux pumps should not be surprising given that most, if not all, bacteria have multiple PGP homologs among their ABC transporters (57–61). Mtb is no exception, with many of its ABC transporters having sequence similarity to human ones including to PGP (62). PGP inhibitors verapamil and reserpine have been found to inhibit the bacterial ABC transporters that were tested (63–65). Our work extends the PGP link to bacterial proton gradient dependent transporters. This link too is unsurprising as homologies exist between bacterial efflux pumps of different classes – for instance the ABC transporter LmrA and the RND transporter MexB (66). Moreover, there is overlap between substrates and inhibitors of efflux pumps of different classes, consistent with the energy sources of the pumps being unrelated to their substrates and inhibitors (67, 68). For example, the MFS pump BmrA of *Bacillus subtilis* transports diverse cationic compounds, including many PGP substrates - fluoroquinolones, ethidium bromide, puromycin and doxorubicin (69). The RND pump AcrB transports all these and also neutral compounds such as chloramphenicol (68). Furthermore, all three pumps can be inhibited by reserpine (64, 70, 71). Identification of the specific Mtb pumps that are inhibited by verapamil and the other PGP inhibitors will further understanding of this complex area.

Our findings suggest that verapamil is a direct inhibitor of PGP and bacterial drug efflux pumps, a conclusion that is well supported by functional data (64, 68, 72–74). Structural data support the functional findings that verapamil inhibits efflux pumps directly, occupying the substrate binding sites of two bacterial MATE transporters (75). Similarly, solid-state nuclear magnetic resonance studies reveal that verapamil penetrates into the substrate-binding cavity of PGP (76) and inhibits it through specific binding interactions therein (74, 76–82). However, a more recent paper by Chen et al. (55), has challenged this mechanism of action for Mtb, reporting that verapamil instead inhibits Mtb efflux by inserting into its membrane, causing depolarization and disrupting proton gradients (55). This result is puzzling because low concentrations of amines with a high pKa (i.e., fully charged at physiological pH) cannot cross membranes unless the charge is highly delocalized (83), and verapamil has an amino group with a pKa ~ 8.8 (84) and exhibits no charge delocalization. The paper concluding that verapamil depolarizes the mycobacterial membrane presents some difficulties because this conclusion was based on measuring membrane potential with a dye, the binding of which could be directly affected by verapamil, rather than accurately reporting the membrane potential.

Moreover, CCCP which was used as a positive control for depolarization failed to show any effect, whereas it showed robust activity in our work at a lower concentration than used in that study (6.25 μM versus 16 μM)(Fig. 2A,B) (55). Finally, it is puzzling that in that study, membrane depolarization, which would be expected to cause inhibition of drug efflux, is cited as both the reason both for verapamil’s activity and for the lack of detection of increased rifampicin accumulation due to verapamil. The latter finding directly contradicts work using a radiolabeled assay showing that verapamil increases accumulation of rifampicin in Mtb (56).

Germane to our work, we note that the conclusions of Chen et al., that verapamil acts as a membrane depolarizer of the Mtb membrane, thereby killing the organisms, are based on its use at far higher concentrations than those used in our assays. The concentrations of verapamil required to kill exponential phase, stationary phase and starved Mtb were 512 μM. Their reported effects of verapamil on membrane polarity as determined by DiSC3 dye were first discerned at 32μM and became substantial from 128μM upwards (55). In contrast, we find that verapamil’s inhibition of FITC-rifampicin efflux starts as low as 5μM and on EtBr from 10μM. Thus, whatever the opinion of the validity of the experiments by Chen et al, it seems likely that our work reports on a different phenomenon, one which is taking place at more biologically relevant concentrations of verapamil. Rather, our work adds to the body of work contradicting the model of verapamil as a membrane depolarizer and provides two further lines of evidence that verapamil acts through direct inhibition of Mtb drug efflux pumps (7, 8, 10). Firstly, the verapamil analogs we have tested all retain the amino group that would be expected to confer the membrane depolarizing activity, yet have different strengths of inhibition of Mtb efflux, 3-fold for rifampicin and >100-fold for EtBr. Secondly, individual analogs inhibit EtBr and rifampicin efflux very differently, by as much as 69-fold in one instance, which would not be the case if verapamil acted simply by disrupting the proton gradient by inserting into membranes. The presence of a negatively charged sidechain in amino acid residues in the transmembrane region of several drug efflux pumps strongly suggests a binding site for a positively charged entity such as a protonated nitrogen, such as found in verapamil. This binding site in human PGP has been analyzed (40–46) and is likely very similar to binding sites in bacterial efflux pumps.

Our findings from the EtBr and verapamil assays provide some insight about relevance in vivo. Our initial analyses with verapamil, its R and S enantiomers and its metabolite, norverapamil as well as piperine and thioridazine suggested that the two assays were equivalent. This would favor the use of EtBr rather than rifampicin as the screening substrate as the EtBr assay is simpler and does not require the synthesis of FITC-rifampicin. However, testing the verapamil analogs revealed that while most compounds inhibited efflux of both albeit often with different strengths, there were outliers that inhibited efflux of one substrate but not the other. Compounds 5D and 11, which had no discernible inhibitory activity against EtBr had strong inhibition against rifampicin efflux. This pattern continued when we tested the diverse panel of PGP-inhibiting drugs, where the PPIs with the strongest inhibition of rifampicin had very little activity against EtBr, and hence would have been missed by this screen alone. Conversely, atorvastatin with the second highest inhibitory activity against EtBr efflux had very little effect against rifampicin efflux. Our finding that the PPIs inhibit intracellular tolerance and growth highlights the importance of the rifampicin efflux assay. We would need to test atorvastatin in vivo to see if the EtBr assay also identifies relevant compounds.

We focused our intracellular validation on the PPIs in large part because they are extremely widely used and well tolerated drugs. These drugs comprise substituted benzimidazole rings which react with the H^+^/K^+^ ATPase in gastric parietal cells to inhibit acid secretion. Protonation of the pyridine and benzimidazole nitrogen results in the formation of a tetracyclic sulfonamide, which binds covalently to exposed cysteine residues in the target (85). We speculate that a similar mechanism could account for its activity against Mtb efflux pumps, with protonation occurring in the bacterial periplasm before binding of the resultant reactive thiol group within the efflux pump channel.

We note that a metabolite of the PPI lansoprazole, lansoprazole sulfide, has been shown to have anti-tuberculous activity via inhibition of the *b*-subunit of the cytochrome *bc*_1_ complex of Mtb (86). However, as lansoprazole sulfide is only formed by metabolic activity of the macrophage itself (85, 86), it cannot account for the inhibition of rifampicin efflux in broth culture. Additionally, the screen which identified lansoprazole found no activity for omeprazole or pantoprazole. In contrast, we find that all four PPIs tested inhibit rifampicin efflux, intramacrophage growth, and macrophage-induced tolerance. Thus, lansoprazole inhibits rifampicin efflux pumps independent of its metabolite’s activity against cytochrome *bc*_1_.

PPIs, widely used over the counter medications worldwide, are associated with very few adverse effects. A study of lansoprazole pharmacokinetics in rats shows that it is concentrated in lung tissue (87), a relevant attribute for treatment of lung TB, the predominant form. In humans, the peak serum concentration (C_max_) of the proton pump inhibitors after a week of oral lansoprazole at standard dosage is around 2.5μM (88), within the range that inhibits rifampicin efflux in our model system. C_max_ values vary depending on dose, duration and study methodology and range from 0.23-23.2μM for omeprazole, 2.87-8.61μM for pantoprazole, and 1.14μM for rabeprazole at a sub-maximal oral dose, again within the range that inhibits rifampicin efflux (89).

The pharmacokinetics of the PPIs in combination with TB drugs will however require evaluation. All of the PPIs except rabeprazole are metabolized to some extent via the P450 cytochrome isoenzyme CYP2C19 (85). Because rifampicin is a strong inducer of hepatic CYP450 metabolism, and would thus result in their increased clearance, increased dosing would be required if used with rifampicin-containing regimens (90). Rabeprazole is metabolized mainly via a nonenzymatic reduction to a thioether compound with relatively minor CYP2C19 and CYP3A4 involvement and therefore may require little or any dosing adjustment when used with rifampicin (85, 91).

Our finding that PPIs limit Mtb intramacrophage growth in the absence of antibiotics raises the question of whether existing epidemiological data point to their protective effect against TB. This is a difficult question from incidental studies, particularly in TB high burden countries. The available data are conflicting: three case-control studies identified an association between PPI use and new diagnosis of pulmonary TB (92–94). A fourth identified an association but only with initial treatment, which inexplicably disappeared after three months of PPI prescription. A fifth study identified a protective effect against TB for lansoprazole when compared to omeprazole and pantoprazole (95). These links are tenuous and must be interpreted with caution. Patients on PPIs are overall more in contact with healthcare systems, and have more of the comorbidities that predispose to TB (93, 94, 96). Reflux esophagitis, one of the common indications for PPI prescription, is itself a risk factor for TB infection (92). Symptoms of gastro-esophageal reflux disease or esophagitis overlap with the symptoms of early TB, which could result in misdiagnosis or over-prescription of PPIs in TB patients (97). Prospective trials would be required to determine whether PPI use reduces TB risk, and whether the adjunctive use of PPIs can shorten TB treatment. As have been done for verapamil (56), studies in the mouse model of TB can determine if PPIs can prevent relapse after shorter treatment. Furthermore, before use in rifampicin-containing regimens, PPI pharmacokinetics must be determined in the presence of rifampicin to identify the correct dose.

Macrophage-induced tolerance develops to almost all known TB drugs, including moxifloxacin, which forms the basis of an emerging new 4 month regimen (4). It will be interesting to see if the PPIs and the other drugs identified in our efflux assays will have a salutary effect in inhibiting macrophage-induced tolerance to other TB drugs. Further, because of the emerging evidence linking drug tolerance and resistance, it is conceivable that adjunctive treatment of TB with PPIs could reduce the emergence of drug resistant strains (98). Finally, it will be important to test their efficacy in inhibiting drug efflux pump mediated growth and tolerance in other pathogenic mycobacteria such as *Mycobacterium avium* and *Mycobacterium abscessus*, which are more recalcitrant to treatment than Mtb.

## Materials and Methods

Verapamil, norverapamil, R-verapamil and S-verapamil were donated by Cipla. The novel verapamil analogues were provided by Prof. Silvia Dei. All other drugs were purchased from Sigma or Cayman unless otherwise stated. Statistical analysis was carried out in Graphpad Prism 9.4.0. Figure S1 was created in BioRender.

### Bacterial Strains, Methods and Culture

*M. tuberculosis ΔleuΔpanCD* (mc^2^6206) (99) was a gift from William Jacobs. *M. tuberculosis* H37Rv (ATCC 27294) was a gift from Christopher Sassetti. An H37Ra Rv1258c knockout strain was constructed from the parental H37Ra strain by specialized phage transduction as per Larsen et al 2007 (100). Deletion of Rv1258c was confirmed by PCR amplification with primer pairs acttcgaggtgttcgaggag, tacgttccaacacatccagcg and catgcaagctcaggatgtcc, atcatcagattccgctcctcg and sequencing. *M. tuberculosis* CDC1551::tnRv1258c (JHU1258c-833), harboring a transposon insertion at position 833 in the Rv1258c ORF, and the wild-type parent strain CDC1551 were from W.R. Bishai and G. Lamichhane (Johns Hopkins University) (101). Mtb strains were grown without agitation at 37°C in Middlebrook 7H9 medium (Difco) supplemented with 10% Middlebrook OADC supplement (Becton Dickinson) with 0.05% Tween-80 unless otherwise stated, or on Middlebrook 7H10 agar (Millipore) with 10% OADC. Growth media for Mtb mc^2^6206 was additionally supplemented with 24 μg/mL pantothenic acid, and 50 μg/mL L-leucine. Cultures were routinely checked for contamination by streaking 20 μL onto Luria-Bertani plates and incubating plates for 48 hours at 37°C. Culture density and growth phase was monitored by measurement of optical density at 600nm (OD_600_) in a 10mm path length cuvette using a spectrophotometer (Eppendorf Biospectrometer).

### Ethidium bromide (EtBr) accumulation assay

Adapted from Rodrigues *et al* (20). Assays were carried out in modified Sauton’s media (m-Sauton’s) with 0.05% Tween-80 (102), without pantothenic acid or L-leucine supplementation, from which ferric ammonium citrate and zinc sulfate were omitted to avoid optical interference and for convenience respectively. Mid-logarithmic cultures between OD_600_ 0.4-0.8 were harvested in 50mL Falcon tubes by centrifugation for 9 minutes at 3220 x *g*, washed and resuspended at OD_600_ 0.4-0.8 in m-Sauton’s, then aliquoted into a 96-well black optical-bottom plate (Costar) in triplicate with drug or vehicle as appropriate. Ethidium bromide (EtBr) (Fisher Scientific, 10132863) in m-Sauton’s was added to each well to a final concentration of 1μg/mL (25% of EtBr’s reported MIC for H37Rv (103). Intrabacterial accumulation of EtBr was measured *in situ* using a plate reader (CLARIOstar, BMG LabTech) by fluorimetry at 530±25 nm excitation and 590±20 nm emission every 60-120 seconds. Measurements were continued until steady state was reached, typically after 90 minutes. For ease of comparison between drugs and experiments, the mean final 10 values measured in steady state under any one condition were expressed as a percentage of the mean final 10 values measured in steady state without drug present.

### Quantification of FITC-rifampicin efflux

In contrast to the EtBr assay, the FITC-rifampicin assay requires intermittent sampling to measure supernatant fluorescence level. M-Sauton’s (as above) was used as a wash buffer throughout. Mid-log phase bacteria were harvested by centrifugation as above, resuspended in m-Sauton’s at OD_600_ 0.8-1.5, and then incubated for 10 minutes at 37 °C. To load the bacteria with FITC-rifampicin, FITC-rifampicin in DMSO was added to a final concentration of 2 μM and cells incubated at 37°C for 30 minutes. To remove free FITC-rifampicin, cells were chilled for 10 minutes in an ice bath, washed three times in 50 mL buffer at 4°C and then resuspended on ice to OD_600_ 0.6-1.0. Drug or vehicle was added, and then the cells were transferred to a 37°C water bath at the start of the time course. If required, an aliquot was then removed and heat-killed at 90°C before being returned to 37°C incubation. Supernatant fluorescence was analyzed at timepoints from bacterial suspensions by vortexing briefly, then passing a 300 μL aliquot through a 0.2 μm syringe filter (Pall Acrodisc) to remove bacteria. Alternatively, where large numbers of different drug treatments were tested simultaneously, 10μL drug or vehicle was aliquoted at 100-fold the required concentration to wells of a 2mL v-bottom deep well 96 well plate (Greiner Bio-one), then 990μL mL bacterial suspension was added and mixed by multichannel. Plates were sealed with a Biorad adhesive micro-seal and incubated at 37°C. At each time point, a single plate was centrifuged for 9 minutes at 3220 x *g*, and then 200 μL supernatant carefully removed without disturbing the pellet. FITC-rifampicin levels in each supernatant were quantified in triplicate by fluorimetry in black, optical bottom half-area 96 well plates (Greiner) using a plate reader (CLARIOstar, BMG LabTech) set to 483±14 nm excitation and 530±30 nm emission. For convenience, a standard 24-hour final sampling time was adopted. For ease of comparison between drugs and experiments, the 24-hour supernatant fluorescence value under any one condition was expressed as a percentage of the mean 24-hour fluorescence value in the untreated group.

### Rifampicin and FITC-rifampicin efflux competition

Mid-log phase bacteria were harvested and prepared as above. FITC-rifampicin was added to a final concentration of 2 μM, and then the bacterial suspension divided into two samples, which were treated either with 4 μM unlabeled rifampicin in DMSO, or DMSO alone. After incubation at 37°C for 30 min, free FITC-rifampicin was removed and supernatant FITC-rifampicin levels quantified as above.

### Measurement of bacterial ATP levels

Mid-log phase bacteria were exposed to drug for 24 hours at 37°C then 25μL of bacteria was mixed with 25μL of BacTiter Glo™ reagent (Promega) as per manufacturer’s instructions and as previously described (104). Samples were mixed by orbital shaking at 100 rpm for 10 minutes at room temperature and then luminescence was measured in a black half-area 96 well plate using a plate reader (CLARIOstar, BMG LabTech) at 545-50 nm emission.

### Minimum Inhibitory Concentration (MIC) Assays

We used a method adapted from Palomino *et al* (105). A two-fold dilution series of drug at 2-fold the desired concentration was prepared in 100 μL volumes of 7H9 plus OADC without Tween-80 in a sterile flat-bottomed 96 well plate (Costar). A bacterial suspension at 10^5^ colony forming units (CFU) per mL was prepared from a mid-logarithmic bacterial suspension in 7H9 plus OADC without Tween-80, then added to wells in 100 μL volumes. Each plate included growth control wells and drug-only negative controls. Parafilm-wrapped plates were incubated for 7-10 days until sufficient growth was observed in control wells by microscopy at 10x magnification using an AE2000 inverted microscope. then 30 μL of resazurin 0.02% + Tween-80 (7.7%) was added overnight. After 24 hours, levels of the fluorescent product of resazurin, resorufin, were measured using a plate reader (CLARIOstar, BMG LabTech) at 530±15 nm excitation, 590±20 nm emission. The MIC was defined as the lowest concentration of drug preventing 95% of resorufin signal.

### Macrophage growth and infection

THP-1 cells (ATCC) were cultured in Roswell Park Memorial Institute 1640 (RPMI) medium, supplemented with 10% fetal calf serum, 10mM HEPES, 1.0mM sodium pyruvate, 0.05mM 2-mercaptoethanol and 2 mM L-glutamine in a 37°C incubator with 5% CO2. In each well of a sterile 24-well plate, 5 x 10^5^ cells per well were differentiated with 100 nM phorbol 12-myristate 13-acetate (PMA) for 48 hours, washed once, then media replaced. After 24 hours rest, cells were infected at a multiplicity of infection (MOI) of 1, incubated for 2-4 hours, and then washed 3 times with PBS before replacing with fresh supplemented RPMI media containing 6 μg/mL streptomycin. For intramacrophage growth assays, streptomycin treatment was discontinued at the time of adding drug. At given timepoints, triplicate wells of infected cells were washed twice with PBS and incubated with 100 μL of 0.1% Triton X-100 for 10 minutes before adding 900 μL of PBS and scraping wells with a pipette tip. A serial dilution of 100 μL of each cell lysate was made in PBS and plated on 7H10 agar to enumerate CFU. Inhibitors were tested for toxicity to macrophages using exposure to resazurin as above with incubation for 4-6 hours. For tolerance assays, triplicate wells were washed briefly with PBS and then with water. Cells were then incubated with 100 μL of water per well at 37°C for 15 minutes. Then 900 μL of 7H9 medium (supplemented with Middlebrook ADC and 0.05% Tween-80) was added, and the wells scraped with a pipette tip. Lysates taken at 2h or 96h were then incubated for a further 48h in the presence of drug. Lysates were plated for enumeration of CFU as described above, at the time of lysis and after 48h further under drug exposure.

## Supplementary Material

Supplementary Material

## Significance Statement

Tuberculosis requires long treatment because of the development of antimicrobial tolerance by *Mycobacterium tuberculosis*. Tolerance develops early when mycobacteria infect macrophages and has been attributed to the induction of bacterial drug efflux pumps upon macrophage residence. Here, we show that drug tolerance is due to proton gradient dependent *M. tuberculosis* efflux pumps that are inhibited by verapamil and other P-glycoprotein inhibitors, including proton pump inhibitors. Our work develops a facile platform for future drug discovery and for the development of proton pump inhibitors or their analogues with potential anti-TB treatment shortening potential.
