# The human proton pump inhibitors inhibit *Mycobacterium tuberculosis* rifampicin efflux and macrophage-induced rifampicin tolerance

**DOI:** 10.1073/pnas.2215512120

**Published:** 2023-02-10

**Authors:** M. Alexandra Lake, Kristin N. Adams, Feilin Nie, Elaine Fowler, Amit K. Verma, Silvia Dei, Elisabetta Teodori, David R. Sherman, Paul H. Edelstein, David R. Spring, Mark Troll, Lalita Ramakrishnan

**Affiliations:** ^a^Molecular Immunity Unit, Cambridge Institute of Therapeutic Immunology and Infectious Diseases, Department of Medicine, University of Cambridge, CB2 0AW Cambridge, UK; ^b^Medical Research Council Laboratory of Molecular Biology, CB2 0QH Cambridge, UK; ^c^Department of Microbiology, University of Washington, Seattle 98195; ^d^Yusuf Hamied Department of Chemistry, University of Cambridge, Cambridge CB2 1EW, UK; ^e^Department of Neuroscience, Psychology, Drug Research and Child Health - Section of Pharmaceutical and Nutraceutical Sciences, University of Florence, 50019 Sesto Fiorentino (FI), Italy; ^f^Perelman School of Medicine, University of Pennsylvania, Philadelphia, PA 19104

**Keywords:** tuberculosis rifampicin tolerance, mycobacterial efflux pumps, efflux pump inhibitors, proton pump inhibitors, verapamil

## Abstract

Tuberculosis treatment requires months-long combination chemotherapy with multiple drugs, with shorter treatments leading to relapses. A major impediment to shortening treatment is that *Mycobacterium tuberculosis* becomes tolerant to the administered drugs, starting early after infection and within days of infecting macrophages. Multiple lines of evidence suggest that macrophage-induced drug tolerance is mediated by mycobacterial drug efflux pumps. Here, using assays to directly measure drug efflux, we find that *M. tuberculosis* transports the first-line antitubercular drug rifampicin through a proton gradient-dependent mechanism. We show that verapamil, a known efflux pump inhibitor, which inhibits macrophage-induced rifampicin tolerance, also inhibits *M.tuberculosis* rifampicin efflux. As with macrophage-induced tolerance, the calcium channel-inhibiting property of verapamil is not required for its inhibition of rifampicin efflux. By testing verapamil analogs, we show that verapamil directly inhibits *M. tuberculosis* drug efflux pumps through its human P-glycoprotein (PGP)-like inhibitory activity. Screening commonly used drugs with incidental PGP inhibitory activity, we find many inhibit rifampicin efflux, including the proton pump inhibitors (PPIs) such as omeprazole. Like verapamil, the PPIs inhibit macrophage-induced rifampicin tolerance as well as intramacrophage growth, which has also been linked to mycobacterial efflux pump activity. Our assays provide a facile screening platform for *M. tuberculosis* efflux pump inhibitors that inhibit in vivo drug tolerance and growth.

## Significance

Tuberculosis requires long-term treatment because of the development of antimicrobial tolerance by *Mycobacterium tuberculosis*. Tolerance develops early when mycobacteria infect macrophages and has been attributed to the induction of bacterial drug efflux pumps upon macrophage residence. Here, we show that drug tolerance is due to proton gradient-dependent *M. tuberculosis* efflux pumps that are inhibited by verapamil and other P-glycoprotein inhibitors, including proton pump inhibitors. Our work develops a facile platform for future drug discovery and for the development of proton pump inhibitors or their analogs with potential anti-TB treatment shortening potential.

Tuberculosis requires months-long multidrug treatment for reliable cures (1), with shorter regimens leading to increased rates of relapse (1–4). Because relapses typically involve genetically drug-susceptible organisms, they are attributed to recrudescence of residual organisms with phenotypic drug resistance, also known as tolerance (5). Drug-tolerant organisms do not have genetic drug resistance mutations and are killed normally under standard laboratory conditions but poorly within the host (5).

Mtb drug tolerance has long been attributed to metabolically quiescent or dormant populations (6). In recent years, we have uncovered a distinct mechanism for Mtb drug tolerance where bacteria become tolerant to multiple antitubercular drugs upon entering macrophages (7). In contrast to the dormant Mtb tolerance models (6), macrophage-induced tolerance is enriched in actively growing bacteria (7). Importantly, macrophage-induced tolerance occurs in diverse Mtb clinical isolates from all four common lineages (8).

Two lines of evidence suggest that macrophage-induced drug tolerance is mediated by Mtb drug efflux pumps. In the virulent laboratory strain, CDC1551, genetic knockdown of the major facilitator superfamily (MFS) efflux pump Tap (Rv1258c), which is transcriptionally induced when Mtb infects macrophages (9), causes loss of macrophage-induced rifampicin tolerance. Consistent with this finding, among clinical Mtb isolates, only the lineage 2 Beijing strains with a loss-of-function mutation in Rv1258c fail to develop macrophage-induced rifampicin tolerance (8). In the second line of evidence, macrophage-induced drug tolerance is inhibited by multiple structurally distinct drugs that are known to inhibit bacterial efflux pumps (7, 10, 11). Rv1258c also constitutes a virulence determinant, promoting Mtb intramacrophage growth in the absence of antibiotics and likewise, the efflux-blocking drugs inhibit intramacrophage Mtb growth in the absence of antibiotics (7, 8, 10). Verapamil, the best studied of these drugs, was shown to decrease relapse rates in mice infected with drug-sensitive TB and given shortened courses of standard antitubercular treatment (12). Furthermore, verapamil reduced bacterial loads in mice infected with a multidrug-resistant Mtb strain (i.e., rifampicin and isoniazid resistant) treated with the standard first-line rifampicin-containing regimen (13).

Here, we develop and validate assays to directly measure Mtb rifampicin efflux and link it to macrophage-induced rifampicin tolerance and intramacrophage growth in the absence of antibiotics. We leverage these assays to identify the PPIs as a new class of approved drugs that inhibit Mtb macrophage-induced tolerance. Finally, our findings support a large body of work finding that verapamil acts directly to inhibit bacterial efflux pumps.

## Results

### Verapamil Inhibits Rifampicin Efflux from Axenically Grown Mtb.

**Fig. 1.**
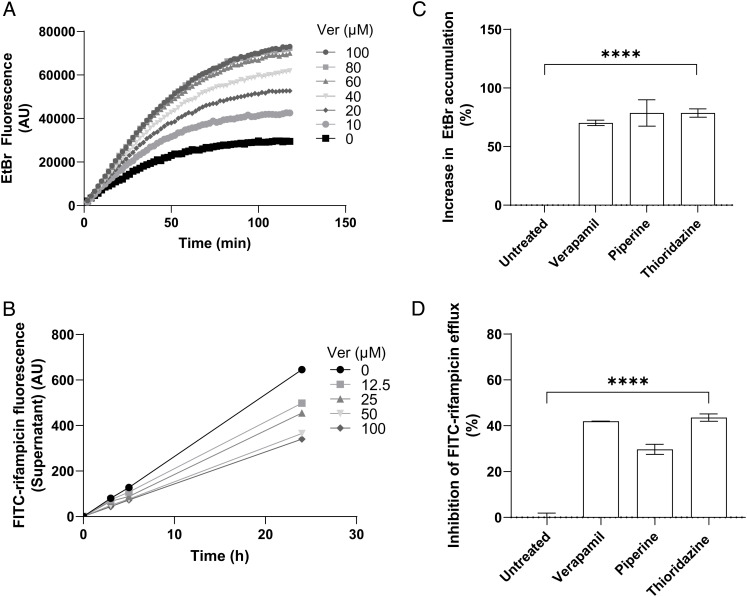
Inhibitors of macrophage-induced Mtb tolerance also inhibit efflux of EtBr and FITC-rifampicin in axenically grown Mtb. (*A*) Mtb mc^2^6206 intracellular EtBr fluorescence over time in the presence of verapamil (Ver) 0 to 100 μM. Values represent means of three technical replicates. (*B*) Efflux of FITC-rifampicin into supernatant over time from Mtb mc^2^6206 treated with Ver 0 to 100 μM. (*C*) Percent increase in intracellular EtBr accumulation in Mtb mc^2^6206 due to Ver 160 μM, piperine 350 μM, or thioridazine 5.4 μM, normalized to mean untreated value. (*D*) Percent inhibition of FITC-rifampicin efflux from Mtb mc^2^6206 due to Ver 160 μM, piperine 350 μM, or thioridazine 5.4 μM, normalized to mean untreated value. (*B*–*D*) Values represent means of three technical replicates ± SEM. Statistical analysis by one-way ANOVA with Dunnett’s multiple comparisons test. **** = *P* < 0.0005.

We used two assays to assess efflux from axenically grown Mtb, using the H37Rv mc^2^6206 strain, a genetically defined leucine and pantothenic acid auxotroph derived from the commonly used virulent strain H37Rv suitable for biosafety level 2 facilities (14–18). In the first assay, we measured ethidium bromide (EtBr) accumulation as a surrogate for rifampicin efflux. The EtBr accumulation assay uses the artificial efflux pump substrate EtBr, the fluorescence of which increases 20-fold when intercalated with DNA (19). Thus, when its efflux is blocked, its accumulation inside bacterial cells, including mycobacteria, renders them more fluorescent (*SI Appendix*, Fig. S1*A*) (20–23). Fluorescence can be measured continuously in a plate reader and typically rises from baseline over 90 to 180 min until a stable steady state is reached between influx and efflux, and fluorescence levels cease to change. Using this assay, we found the expected time-dependent increase in bacterial fluorescence, indicating progressive EtBr accumulation. As shown before (22, 24–26), verapamil increased EtBr accumulation in a concentration-dependent manner at concentrations as low as 5% of its minimum inhibitory concentration (MIC) for H37Rv mc^2^6206 (Fig. 1*A* and *SI Appendix,* Table S1). These concentrations were also within the range of those required to inhibit macrophage-induced rifampicin tolerance (10).

To specifically assay rifampicin efflux, we used rifampicin conjugated to the fluorophore fluorescein isothiocyanate (FITC). We chose FITC for its relatively small size and used a long, flexible carbon linker to minimize its steric hindrance (*SI Appendix*, Fig. S2*A* and *Supplementary Methods*). The FITC-rifampicin conjugate retained some activity against Mtb (MIC 16-fold the rifampicin MIC) (*SI Appendix,* Table S1), comparable to the ninefold MIC increase reported for a different FITC-rifampicin (27). To measure rifampicin efflux, we loaded bacteria with FITC-rifampicin at 37 °C for 30 min, washed at 4 °C to remove conjugate adhering to bacterial surfaces, and then measured supernatant fluorescence over time at 37 °C. In contrast to the EtBr accumulation assay, measurement was not continuous but by intermittent sampling to separate supernatant from bacteria for analysis (*SI Appendix,* Fig. S1*B*). Supernatant fluorescence increased over the assay period, indicating time-dependent efflux (*SI Appendix,* Fig. S2*B*). To ensure that our assay was measuring active rifampicin transport rather than passive diffusion, we performed the assay at 4 °C, which should inhibit active transport but not passive diffusion (28). No significant rifampicin efflux was observed from Mtb maintained at 4 °C, confirming that our assay is a measure of active transport (*SI Appendix,* Fig. S2*B*). To confirm that FITC-rifampicin is transported by the same route as rifampicin, we set up a competition assay by loading cells with 2 μM FITC-rifampicin and 4 μM unlabeled rifampicin in the assay. The unlabeled rifampicin significantly decreased FITC-rifampicin efflux at 90 min, suggesting that FITC-rifampicin is transported by the same efflux pumps as rifampicin (*SI Appendix*, Fig. S2*C*). While EtBr accumulation reaches a final steady state level within 1 to 2 h, FITC-rifampicin efflux continued for at least 24 h. For ease of comparison between assays, we adopted a final 24 h end point in the FITC-rifampicin assay.

Verapamil reduced FITC-rifampicin efflux in a concentration-dependent manner down to 5 μM (Fig. 1*B* and *SI Appendix,* Fig. S3*A*); these concentrations were similar to those inhibiting EtBr efflux (Fig. 1*A*). Verapamil had no effect on the efflux of unconjugated fluorescein, showing that its reduction of FITC-rifampicin efflux is specifically due to its effect on rifampicin efflux (*SI Appendix,* Fig. S3*B*). Next, we tested two other drugs, piperine and thioridazine, which inhibit bacterial efflux pumps in vitro and macrophage-induced rifampicin tolerance (10, 11, 29). As shown before, both drugs inhibited EtBr efflux (26, 30) (Fig. 1*C*). They also inhibited rifampicin efflux (Fig. 1*D*). All three drugs were used at concentrations that do not inhibit Mtb growth over 48 h (10). Nevertheless, to ensure that any observed reductions in FITC-rifampicin efflux were not due to microbicidal effects, we performed the assay on heat-killed Mtb. In contrast to the effects of the compounds on live bacteria, loss of Mtb viability increased FITC-rifampicin release into the medium, with the addition of verapamil (25 μM) having no further effect (*SI Appendix*, Fig. S2*D**.*) This result is consistent with nonviable Mtb having decreased cell wall integrity and confirms the specificity of the assay. In sum, three structurally distinct compounds inhibit both rifampicin efflux from axenically grown Mtb and efflux-pump mediated rifampicin tolerance that develops when Mtb becomes macrophage resident. This finding suggested that pumps with overlapping substrates and inhibitors mediate in vivo and in vitro efflux.

### Rifampicin Efflux Is Mediated Principally by Proton Gradient-Dependent Transporters.

**Fig. 2.**
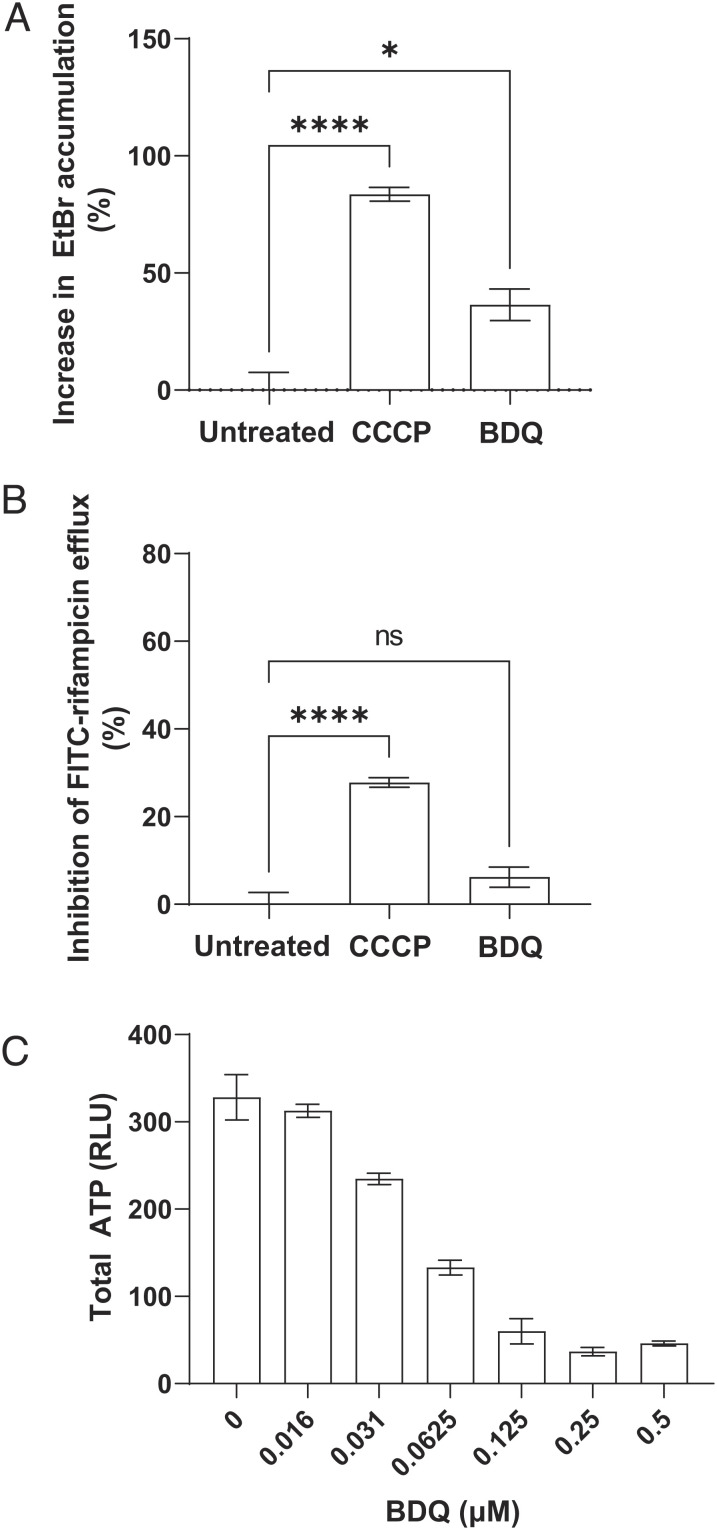
Rifampicin efflux is mediated principally by proton gradient-dependent transporters (*A*) Percent increase in Mtb mc^2^6206 intracellular EtBr accumulation due to CCCP 6.25 μM or bedaquiline (BDQ) 0.0625 μM (25% of MIC). (*B*) Percent inhibition of FITC-rifampicin efflux from Mtb mc^2^6206 due to CCCP 6.25 μM or BDQ 0.0625 μM. (*C*) Total Mtb mc^2^6206 ATP after 24 h treatment with BDQ. RLU, relative luminescence units. Values represent mean of three technical replicates ± SEM. **** = *P* < 0.0005. Representative of at least two experiments at similar concentrations. Statistical analysis by one-way ANOVA with Dunnett’s multiple comparisons test.

Most bacterial, including mycobacterial, drug efflux pumps belong to one of the four major classes: the adenosine triphosphate (ATP)-binding cassette (ABC) transporter class powered by ATP hydrolysis with the remaining three, the MFS, resistance-nodulation-division (RND), and small multidrug resistance (SMR) group pumps being ATP independent and exclusively dependent on the membrane proton gradient for transport (11). Many mycobacterial efflux pumps that are macrophage-induced, or are involved in virulence, belong to the ABC family or MFS families, including the MFS transporter Tap/Rv1258c, which mediates macrophage-induced rifampicin tolerance (7, 10). We confirmed that Rv1258c is a major contributor to macrophage-induced rifampicin tolerance – not only was tolerance lost in Rv1258c mutant Mtb but also the addition of verapamil had no additional effect (*SI Appendix,* Fig. S4*A*). However, modulation of Rv1258c expression in axenic culture, where Rv1258c is present at only basal levels (9), has minimal effects on rifampicin susceptibility (31–34). It was therefore unlikely to contribute to the rifampicin efflux observed in these assays. We confirmed this by testing a Rv1258c mutant in the Mtb H37Ra strain, which, like Mtb H37Rv mc^2^6206, is suitable for containment level 2 use. The Tap/Rv1258c mutant exhibited neither altered EtBr accumulation nor FITC-rifampicin efflux and retained verapamil sensitivity similar to the isogenic H37Ra parent strain, suggesting that the low Rv1258c expression in broth did not contribute to rifampicin efflux (*SI Appendix*, Fig. S4 *B* and *C*). We asked whether other proton gradient-dependent transporters were involved by testing the effect of the protonophore carbonyl cyanide m-chlorophenyl hydrazone (CCCP) (35) at 25% of its MIC for H37Rv mc^2^6206 (*SI Appendix*, Table S1). CCCP strongly inhibited both rifampicin and EtBr efflux (Fig. 2 *A* and *B*). In contrast, the ATP synthase inhibitor bedaquiline (35) at 25% of its MIC for H37Rv mc^2^6206 (*SI Appendix*, Table S1) had no significant effect on rifampicin efflux and only a modest effect on EtBr accumulation (Fig. 2 *A* and *B*). We confirmed that bedaquiline showed the expected inhibition of ATP synthesis: it depleted 59.5 ± 2.6% (mean ± SEM) of total bacterial ATP at the concentration used in the efflux assays (Fig. 2*C*). This finding could explain the shared inhibitory activity of the three drugs on efflux in vitro and macrophage tolerance and lend further support for screening for inhibition of EtBr/rifampicin efflux in vitro as an initial step to identify inhibitors of drug tolerance in vivo.

### Verapamil’s Inhibition of Mtb Efflux Pumps Is Linked to Its Human P-glycoprotein (PGP) Inhibitory Activity.

Verapamil has two distinct activities that are dissociable from each other. It inhibits calcium channels, which is the basis of its clinical use as a cardiovascular drug, and it inhibits a major human promiscuous multidrug ABC transporter, PGP (36, 37). We have shown that verapamil inhibits macrophage-induced rifampicin tolerance independent of its calcium channel-blocking activity; its R-enantiomer and its major metabolite norverapamil, both with reduced cardiac activity thought to be due to their lower inhibitory activity of calcium channel blocker in relation to S-verapamil (38, 39), inhibit rifampicin tolerance with similar efficacy to racemic verapamil (10).

**Fig. 3.**
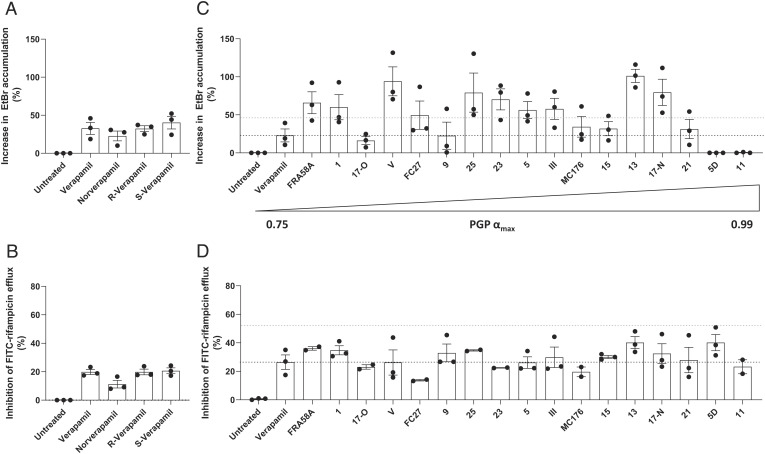
Verapamil analogs targeting PGP increase accumulation of EtBr and inhibit the efflux of FITC-rifampicin in Mtb. (*A* and *C*) Percent increase in intracellular EtBr accumulation in Mtb mc^2^6206 due to 25 μM drug. (*B* and *D*) Percent inhibition of FITC-rifampicin efflux from Mtb mc^2^6206 due to 25 μM drug. (*C* and *D*) Drugs ordered *Left* to *Right* by increasing efficacy of PGP inhibition (α_max_). α_max_ is the maximum increase in the nuclear concentration of the PGP substrate pirarubicin in eukaryotic pirarubicin-resistant cells that can be obtained with a given compound, where α varies between 0 (no inhibitor present) and 1 (when the amount of pirarubicin in resistant cells is the same as in sensitive cells) (40–46). Black dashed line indicates mean value for verapamil; blue dashed line indicates two-fold verapamil activity. (*C* and *D*) Gaussian distribution of data was confirmed by assessment of QQ plot and then outliers within each experiment were detected using Grubbs’ test (α = 0.05) and removed and remaining values were normalized to the mean untreated value. (*A*–*D*) Each symbol represents the mean normalized value for that drug for one individual experiment. (*A*–*C*) Combined data of three experiments. (*D*) Combined data of two (compounds 11, 17-Old, 23, 25, FC27, MC176, FRA58A) or three experiments.

Accordingly, R-verapamil inhibited both EtBr and rifampicin efflux to the same extent as racemic verapamil and its cardiac-active S-enantiomer (Fig. 3 *A* and *B*), suggesting a mechanism unlinked to its calcium channel inhibition, such as PGP inhibition. Norverapamil also inhibited both efflux activities albeit less than the other compounds (Fig. 3 *A* and *B*). However, at higher norverapamil concentrations, which inhibit macrophage-induced tolerance (10), rifampicin efflux was inhibited to a similar degree as verapamil (*SI Appendix*, Fig. S5). This finding was consistent with the inhibition of rifampicin efflux by verapamil being independent of its calcium channel activity, like its inhibition of rifampicin tolerance, and suggested that it was instead linked to its PGP-inhibiting activity.

To probe this, we screened a panel of 17 verapamil analogs which specifically target PGP with negligible cardiac effects, and therefore have minimal calcium channel inhibitory activity (*SI Appendix*, Table S2) (40–46). These compounds were enriched for inhibitors of both EtBr and rifampicin efflux. 15 inhibited EtBr efflux, 10 with two-fold or greater potency than verapamil (Fig. 3*C* and *SI Appendix*, Table S3). All inhibited rifampicin efflux, many with similar or greater (albeit < twofold) efficacy than verapamil (Fig. 3*D* and *SI Appendix*, Table S4). There was no correlation between the strengths of PGP and mycobacterial efflux pump inhibition, which is not surprising given that PGP and bacterial transporters will have structural differences (Fig. 3 *C* and *D* and *SI Appendix* Fig. S6 *A* and *B* and Table S3 and S4). Importantly, there was also not a good correlation for strength of inhibition of EtBr and FITC-rifampicin efflux. Two of the compounds, 5D and 11, had no activity against EtBr efflux and good activity against rifampicin efflux (Fig. 3 *C* and *D* and *SI Appendix F*ig. S6*C*). Thus, distinct bacterial transporters with mostly but not completely overlapping substrate specificities may transport these two substances. Overall, these findings suggested that screening PGP inhibitors for Mtb efflux inhibition can identify new tolerance-inhibiting drugs.

### The Proton Pump Inhibitor (PPI) Class of Drugs Are Potent Inhibitors of Rifampicin Efflux.

**Fig. 4.**
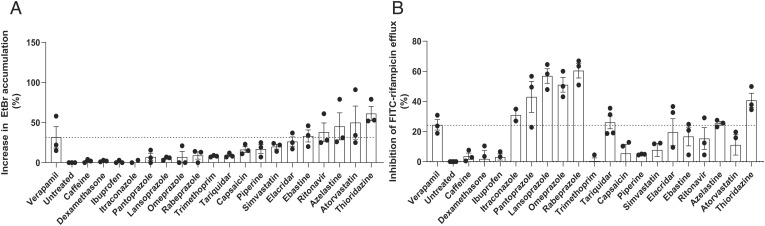
Omeprazole, lansoprazole, rabeprazole, and pantoprazole are potent inhibitors of rifampicin but not EtBr efflux. (*A*) Percent increase in intracellular EtBr accumulation in Mtb mc^2^6206 due to 25 μM drug. (*B*) Percent inhibition of FITC-rifampicin efflux from Mtb mc^2^6206 due to 25 μM drug. (*A* and *B*) Dashed line indicates mean value for verapamil. Gaussian distribution of data was confirmed by assessment of QQ plot and then outliers within each experiment were detected using Grubbs’ test (α = 0.05) and removed, and then remaining values were normalized to the mean untreated value. Each symbol represents the mean normalized value for that drug for one individual experiment. Column height and error bars represent mean ± SEM of all mean values shown for that drug. (*A*) Combined data of three experiments. (*B*) Combined data of three experiments, except for itraconazole, which was tested twice.

PGP inhibition is a well-documented “off-target” effect of many approved drugs (47). We screened a panel of drugs with incidental PGP inhibitory activity for inhibition of Mtb EtBr and rifampicin efflux inhibition, prioritizing inexpensive, widely available drugs using DrugBank (48). This panel was also enriched for inhibition of EtBr and FITC-rifampicin efflux (Fig. 4 *A* and *B* and *SI Appendix*, Tables S3 and S4). As with the verapamil analogs, there was not a correlation in the strength of the compounds for inhibition of efflux of the two substrates (*SI Appendix F*ig. S4*D*). However, there was some concordance in whether they had efflux activity. Five and 10 of the 19 drugs, respectively, inhibited EtBr and rifampicin efflux, with three significantly inhibiting both (Fig. 4 *A* and *B*). One drug, atorvastatin, inhibited EtBr well but was a poor inhibitor of rifampicin efflux (Fig. 4 *A* and *B*). The most striking difference was that the four PPIs, omeprazole, lansoprazole, pantoprazole, and rabeprazole, showed negligible inhibition of EtBr efflux and were the strongest inhibitors of rifampicin efflux (Fig. 4 *A* and *B*) at the screening concentration of 25 μM. Indeed, all the four PPIs inhibited rifampicin efflux even at 2.5 μM, the lowest concentration tested (*SI Appendix,* Fig. S7*A*). This concentration was far (40 to 160-fold) below the MIC of these drugs (*SI Appendix,* Table S1). However, we further confirmed that their effects on rifampicin efflux were specific rather than due to altered bacterial viability affecting cell wall permeability. The fluorescent nuclear dye propidium iodide has increased penetration into killed Mtb due to decreased cell wall integrity, for which it is a sensitive assay (*SI Appendix,* Fig. S7*B*). Using rabeprazole as a representative of the PPI class, we found that it did not cause any increase in propidium iodide uptake after 24 h exposure of FITC-rifampicin-loaded cells up to a concentration of 100 μM, its MIC for Mtb (*SI Appendix,* Fig. S7*B* and Table S1). Together, these findings reinforce our findings that Mtb efflux pump inhibitors are enriched among drugs with incidental PGP-inhibiting activity.

### The PPIs Inhibit Mtb Macrophage-Induced Rifampicin Tolerance and Intramacrophage Growth in the Absence of Antibiotics.

**Fig. 5.**
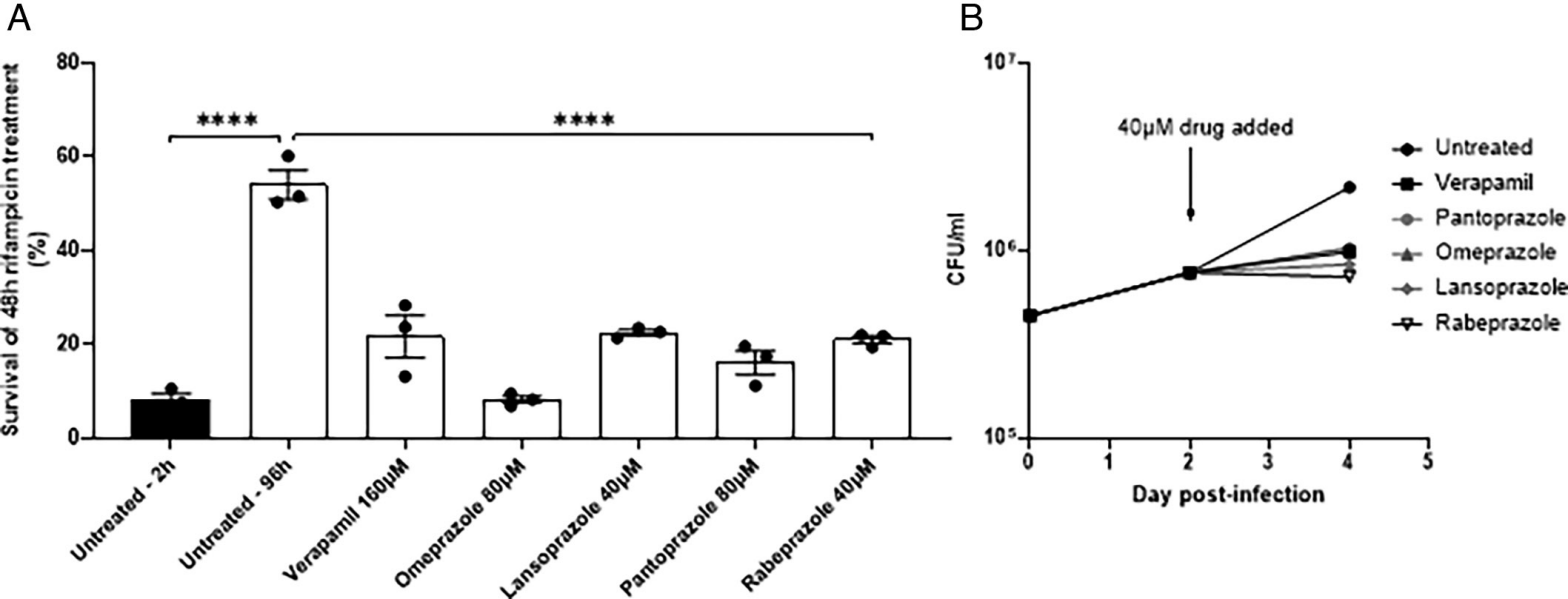
The PPIs inhibit intramacrophage growth and rifampicin tolerance of Mtb. (*A*) THP-1 macrophages were infected with Mtb H37Rv and lysed for 2 h (black bars) or 96 h (white bars) after infection. The released bacteria were treated for an additional 48 h with 1 μg/mL rifampicin with or without inhibitor before enumeration of CFU. Statistical analysis by ordinary one-way ANOVA with Dunnett’s multiple comparisons test. Representative of two independent experiments. (*B*) Growth of Mtb H37Rv in THP-1 macrophages infected at MOI 1. 40 μM drug or vehicle was added at 2 d post-infection and growth allowed to continue a further 48 h. Macrophages were lysed in H_2_O and the lysate was plated in dilution for CFU. Results representative of two or more independent experiments, except lansoprazole 40 μM, which was tested once. (*A* and *B*) Error bars represent SEM. ****P < 0.0005.

Testing verapamil in a direct efflux assay led us to identify PGP-inhibiting drugs as potent inhibitors of rifampicin efflux in axenically grown Mtb. So far, we had validated our in vitro efflux assays by showing that the three drugs we had previously found to inhibit rifampicin drug tolerance in vivo, also inhibited in vitro efflux. We now wanted to go back full circle to determine if the drugs identified in the in vitro assays inhibit intramacrophage rifampicin tolerance. We selected for testing the four PPIs, omeprazole, pantoprazole, lansoprazole, and rabeprazole for two reasons: 1) they are among the most widely used drugs, inexpensive with an excellent tolerability and safety profile (49–51); and 2) their selective inhibition of rifampicin but not EtBr efflux would allow us to test which of the assays was more relevant in vivo. Because Mtb mc^2^6206 is attenuated for growth in THP-1 macrophages (52), we used the parent strain Mtb H37Rv which exhibits macrophage-induced rifampicin tolerance similar to clinical isolates (7, 8, 10). Macrophage-induced Mtb tolerance to antimicrobial agents is defined as the occurrence of reduced antimicrobial killing following macrophage residence (7, 10). Tolerance to rifampicin develops by 96 h of macrophage residence (7, 10). We infected the THP-1 human macrophage cell line with Mtb H37Rv for 96 h, then lysed the cells, and incubated the lysates for 48 h with 1 μg/mL rifampicin (fivefold its MIC for this strain) with or without the individual PPIs (7, 8, 10) (*SI Appendix,* Fig. S8). Verapamil was used at 160 μM (50% of MIC) as before (10). We adjusted the PPI concentrations to be at 25 to 50% of the verapamil concentration and 50% or more below their MICs for H37Rv mc^2^6206 (*SI Appendix,* Table S1). As expected (7, 8, 10), Mtb developed rifampicin tolerance after 96 h of macrophage growth, which was inhibited by verapamil (Fig. 5*A*). All four PPIs inhibited this tolerance similarly (Fig. 5*A*)*.* Verapamil, as well as the other two drugs – thioridazine and piperine – also inhibits Mtb intramacrophage growth in the absence of antimicrobials (7, 8, 10), likely by disrupting their efflux from transporters that transport, in addition to rifampicin, host molecules that are toxic to the bacteria (7, 8, 10). We found that all four PPIs inhibited Mtb intramacrophage growth similarly to verapamil (Fig. 5*B*). Thus, our in vitro rifampicin efflux assay allowed us to identify the PPIs as inhibitors of Mtb macrophage-induced rifampicin tolerance and intramacrophage growth with similar or greater potency than verapamil.

## Discussion

Our whole-cell Mtb efflux assays strengthen the link between rifampicin drug efflux pumps and macrophage-induced drug tolerance, confirm that verapamil inhibits efflux and drug tolerance by direct inhibition of Mtb efflux pumps, and demonstrate the success of a relatively facile platform for screening for inhibitors of in vivo tolerance. We decided to study efflux in axenically grown Mtb because it was not technically feasible to do so from macrophage-resident bacteria – the relatively small numbers of bacteria that can be obtained from macrophages would be insufficient for the assays. We reasoned that axenically grown Mtb would be relevant to in vivo conditions even if the exact pumps mediating efflux in vitro and in vivo were different; for instance, Tap/Rv1258c which mediates macrophage-induced tolerance is expressed at relatively low levels in axenically grown Mtb and is transcriptionally induced in macrophages (9, 53). We were optimistic because verapamil, which inhibits macrophage-induced rifampicin tolerance, is a well-known bacterial efflux pump inhibitor (11) and inhibits EtBr efflux from axenically grown Mtb (2–5) (54). Furthermore, verapamil inhibits macrophage-induced tolerance to rifampicin, isoniazid, and bedaquiline, all likely mediated by distinct efflux pumps (10).Together, these findings suggested that verapamil inhibits different pumps with overlapping and even distinct substrate specificities. We tested this by showing that verapamil inhibits not just EtBr efflux but also rifampicin efflux from axenically grown Mtb. The latter finding is important as previous studies yielded conflicting results. A study using LC/MS to measure rifampicin levels did not detect an increase in its accumulation in Mtb treated with verapamil (55) whereas a study using radiolabeled rifampicin accumulation over one and 2 h found increased intracellular accumulation of ^3^H-rifampicin in the presence of verapamil (12). Further strengthening the link between drug efflux in vitro and in vivo efflux (reflected by macrophage-induced drug tolerance), we find that verapamil inhibits both through its PGP inhibitory rather than calcium channel inhibitory activity. Importantly, PGP-inhibiting drugs discovered through the in vitro efflux assays also inhibit efflux pump-linked mycobacterial growth and rifampicin tolerance in macrophages.

Our finding that many drugs with PGP inhibitory activity inhibit mycobacterial drug efflux pumps should not be surprising given that most, if not all, bacteria have multiple PGP homologs among their ABC transporters (56–60). Mtb is no exception, with many of its ABC transporters having sequence similarity to human ones including PGP (61). PGP inhibitors, verapamil and reserpine, have been found to inhibit the bacterial ABC transporters that were tested (62–64). Our work extends the PGP link to bacterial proton gradient-dependent transporters. This link too is unsurprising as homologies exist between bacterial efflux pumps of different classes – for instance, the ABC transporter LmrA and the RND transporter MexB (65). Moreover, there is overlap between substrates and inhibitors of efflux pumps of different classes, consistent with the energy sources of the pumps being unrelated to their substrates and inhibitors (66, 67). For example, the MFS pump BmrA of *Bacillus subtilis* transports diverse cationic compounds, including many PGP substrates – fluoroquinolones, EtBr, puromycin, and doxorubicin (68). The RND pump AcrB transports all these and also neutral compounds such as chloramphenicol (67). Furthermore, all the three pumps can be inhibited by reserpine (63, 69, 70). Identification of the specific Mtb pumps that are inhibited by verapamil and the other PGP inhibitors will further the understanding of this complex area.

Our findings suggest that verapamil is a direct inhibitor of PGP and bacterial drug efflux pumps, a conclusion that is well supported by functional data (63, 67, 71–73). Structural data support the functional findings that verapamil inhibits efflux pumps directly, occupying the substrate-binding sites of two bacterial MATE transporters (74). Similarly, solid-state NMR studies reveal that verapamil penetrates into the substrate-binding cavity of PGP (75) and studies using multiple techniques show that it inhibits PGP through specific binding interactions therein (73, 75–81). However, a more recent paper by Chen et al. (55) has challenged this mechanism of action for Mtb, reporting that verapamil instead inhibits Mtb efflux by inserting into its membrane, causing depolarization and disrupting proton gradients (55). This result is puzzling because low concentrations of amines with a high pKa (i.e., fully charged at physiological pH) cannot cross membranes unless the charge is highly delocalized (82), and verapamil has an amino group with a pKa ~ 8.8 (83) and exhibits no charge delocalization. The paper concluding that verapamil depolarizes the mycobacterial membrane presents some difficulties because this conclusion was based on measuring membrane potential with a dye, the binding of which could be directly affected by verapamil, rather than accurately reporting the membrane potential. Moreover, CCCP which was used as a positive control for depolarization failed to show any effect, whereas it showed robust activity in our work at a lower concentration than used in that study (6.25 μM versus 16 μM) (Fig. 2 *A* and *B*) (55). Finally, it is puzzling that in that study, membrane depolarization, which would be expected to cause inhibition of drug efflux, is cited as the reason both for verapamil’s activity and for the lack of detection of increased rifampicin accumulation due to verapamil. The latter finding directly contradicts work using a radiolabeled assay showing that verapamil increases accumulation of rifampicin in Mtb (12).

Germane to our work, we note that the conclusions of Chen et al., that verapamil acts as a membrane depolarizer of the Mtb membrane, thereby killing the organisms, are based on its use at far higher concentrations than those used in our assays. The concentrations of verapamil required to kill exponential phase, stationary phase, and starved Mtb were 512 μM. Their reported effects of verapamil on membrane polarity as determined by DiSC_3_ dye were first discerned at 32 μM and became substantial from 128 μM upward (55). In contrast, we find that verapamil’s inhibition of FITC-rifampicin efflux starts as low as 5 μM and on EtBr from 10 μM. Thus, irrespective of the opinion of the validity of the experiments by Chen et al., it seems likely that our work reports on a different phenomenon, one which is taking place at more biologically relevant concentrations of verapamil. Rather, our work adds to the body of work contradicting the model of verapamil as a membrane depolarizer and provides two further lines of evidence that verapamil acts through direct inhibition of Mtb drug efflux pumps (7, 8, 10). First, the verapamil analogs we have tested all retain the amino group that would be expected to confer the membrane-depolarizing activity, yet have different strengths of inhibition of Mtb efflux, threefold for rifampicin and >100-fold for EtBr. Second, individual analogs inhibit EtBr and rifampicin efflux very differently, by as much as 69-fold in one instance, which would not be the case if verapamil acted simply by disrupting the proton gradient by inserting into membranes. The presence of a negatively charged sidechain in amino acid residues in the transmembrane region of several drug efflux pumps strongly suggests a binding site for a positively charged entity such as a protonated nitrogen as is found in verapamil. This binding site in human PGP has been analyzed (40–46) and is likely very similar to binding sites in bacterial efflux pumps.

Our findings from the EtBr and verapamil assays provide some insight about relevance in vivo. Our initial analyses with verapamil, its R and S enantiomers and its metabolite, norverapamil, as well as piperine and thioridazine suggested that the two assays were equivalent. This would favor the use of EtBr rather than rifampicin as the screening substrate as the EtBr assay is simpler and does not require the synthesis of FITC-rifampicin. However, testing the verapamil analogs revealed that while most compounds inhibited efflux of both albeit often with different strengths, there were outliers that inhibited efflux of one substrate but not the other. Compounds 5D and 11, which had no discernible inhibitory activity against EtBr, had strong inhibition against rifampicin efflux. This pattern continued when we tested the diverse panel of PGP-inhibiting drugs, where the PPIs with the strongest inhibition of rifampicin had very little activity against EtBr, and hence would have been missed by this screen alone. Conversely, atorvastatin with the second highest inhibitory activity against EtBr efflux had very little effect against rifampicin efflux. Our finding that the PPIs inhibit intracellular tolerance and growth highlights the importance of the rifampicin efflux assay. We would need to test atorvastatin in vivo to see if the EtBr assay also identifies relevant compounds.

We focused our intracellular validation on the PPIs in large part because they are extremely widely used and well-tolerated drugs. These drugs comprise substituted benzimidazole rings which react with the H^+^/K^+^ ATPase in gastric parietal cells to inhibit acid secretion. Protonation of the pyridine and benzimidazole nitrogen results in the formation of a tetracyclic sulfonamide, which binds covalently to exposed cysteine residues in the target (84). We speculate that a similar mechanism could account for its activity against Mtb efflux pumps, with protonation occurring in the bacterial periplasm before binding of the resultant reactive thiol group within the efflux pump channel.

We note that a metabolite of the PPI lansoprazole, lansoprazole sulfide, has been shown to have antituberculous activity via inhibition of the *b*-subunit of the cytochrome *bc*_1_ complex of Mtb (85). However, as lansoprazole sulfide is only formed by metabolic activity of the macrophage itself (84, 85), it cannot account for the inhibition of rifampicin efflux in broth culture. Moreover, the study which identified lansoprazole found no activity for omeprazole or pantoprazole (86). In contrast, we find that all the four PPIs tested inhibit rifampicin efflux, intramacrophage growth, and macrophage-induced tolerance. Thus, lansoprazole inhibits rifampicin efflux pumps independent of its metabolite’s activity against cytochrome *bc*_1_.

PPIs, widely used as over-the-counter medications worldwide, are associated with very few adverse effects. A study of lansoprazole pharmacokinetics in rats shows that it is concentrated in lung tissue (86), a relevant attribute for treatment of lung TB, the predominant form. In humans, the peak serum concentration (C_max_) of tlanzoprazole after a week of oral administration at standard dosage is around 2.5 μM (87), within the range that inhibits rifampicin efflux in our model system. C_max_ values vary depending on dose, duration, and study methodology and range from 0.23 to 23.2 μM for omeprazole, 2.87 to 8.61 μM for pantoprazole, and 1.14 μM for rabeprazole at a sub-maximal oral dose, again within the range that inhibits rifampicin efflux (88).

The pharmacokinetics of the PPIs in combination with TB drugs will however require evaluation. All of the PPIs except rabeprazole are metabolized to some extent via the P450 cytochrome isoenzyme CYP2C19 (84). Because rifampicin is a strong inducer of hepatic CYP450 metabolism, and would thus result in their increased clearance, increased dosing would be required if used with rifampicin-containing regimens (89). Rabeprazole is metabolized mainly via a nonenzymatic reduction to a thioether compound with relatively minor CYP2C19 and CYP3A4 involvement and therefore may require little if any dosing adjustment when used with rifampicin (84, 90).

Our finding that PPIs limit Mtb intramacrophage growth in the absence of antibiotics raises the question of whether existing epidemiological data point to their protective effect against TB. This is a difficult question from incidental studies, particularly in TB high-burden countries. The available data are conflicting: three case–control studies identified an association between PPI use and new diagnosis of pulmonary TB (91–93). A fourth identified an association but only with initial treatment, which inexplicably disappeared after 3 mo of PPI prescription. A fifth study identified a protective effect against TB for lansoprazole when compared to omeprazole and pantoprazole (94). These links are tenuous and must be interpreted with caution. Patients on PPIs are overall more in contact with health-care systems and have more of the comorbidities that predispose to TB (92, 93, 95). Reflux esophagitis, one of the common indications for PPI prescription, is itself a risk factor for TB infection (91). Symptoms of gastroesophageal reflux disease or esophagitis overlap with the symptoms of early TB, which could result in misdiagnosis or overprescription of PPIs in TB patients (96). Prospective trials would be required to determine whether PPI use reduces TB risk, and whether the adjunctive use of PPIs can shorten TB treatment. As have been done for verapamil (12), studies in the mouse model of TB can determine whether PPIs can prevent relapse after shorter treatment. Furthermore, before use in rifampicin-containing regimens, PPI pharmacokinetics must be determined in the presence of rifampicin to identify the correct dose.

Macrophage-induced tolerance develops to almost all known TB drugs, including moxifloxacin, which forms the basis of an emerging new 4 mo regimen (4). It will be interesting to see whether the PPIs and the other drugs identified in our efflux assays will have a salutary effect in inhibiting macrophage-induced tolerance to other TB drugs. Further, because of the emerging evidence linking drug tolerance and resistance, it is conceivable that adjunctive treatment of TB with PPIs could reduce the emergence of drug-resistant strains (97). Finally, it will be important to test their efficacy in inhibiting drug efflux pump-mediated growth and tolerance in other pathogenic mycobacteria such as *Mycobacterium avium* and *Mycobacterium abscessus*, which are more recalcitrant to treatment than Mtb.

## Materials and Methods

Verapamil, norverapamil, R-verapamil, and S-verapamil were donated by Cipla. All other drugs were purchased from Sigma or Cayman unless otherwise stated. Statistical analysis was carried out in GraphPad Prism 9.4.0. *SI Appendix*, Fig. S1 was created using BioRender.com.

### Bacterial Strains, Methods, and Culture.

*M. tuberculosis ΔleuΔpanCD* (mc^2^6206) (14) was obtained from W.R. Jacobs, Jr. *M. tuberculosis* H37Rv (ATCC 27294) was obtained from C.M. Sassetti. An H37Ra Rv1258c knockout strain was constructed from the parental H37Ra strain by specialized phage transduction as per Larsen et al. (98). Deletion of Rv1258c was confirmed by PCR amplification with primer pairs: acttcgaggtgttcgaggag, tacgttccaacacatccagcg and catgcaagctcaggatgtcc, atcatcagattccgctcctcg and sequencing. *M. tuberculosis* CDC1551::tnRv1258c (JHU1258c-833), harboring a transposon insertion at position 833 in the Rv1258c ORF, and the wild-type parent strain CDC1551 were obtained from W.R. Bishai and G. Lamichhane (Johns Hopkins University) (99). Mtb strains were grown without agitation at 37 °C in Middlebrook 7H9 medium (Difco) supplemented with 10% Middlebrook OADC supplement (Becton Dickinson) with 0.05% Tween-80 unless otherwise stated, or on Middlebrook 7H10 agar (Millipore) with 10% OADC. Growth media for Mtb mc^2^6206 was additionally supplemented with 24 μg/mL pantothenic acid, and 50 μg/mL L-leucine. Cultures were routinely checked for contamination by streaking 20 μL onto Luria–Bertani plates and incubating plates for 48 h at 37 °C. Culture density and growth phase were monitored by measurement of optical density at 600 nm (OD_600_) in a 10-mm path length cuvette using a spectrophotometer (Eppendorf Biospectrometer).

### EtBr Accumulation Assay.

We used a method adapted from Rodrigues et al. (20). Assays were carried out in modified Sauton’s media (m-Sauton’s) with 0.05% Tween-80 (100), without pantothenic acid or L-leucine supplementation, from which ferric ammonium citrate and zinc sulfate were omitted to avoid optical interference and for convenience, respectively. Mid-logarithmic cultures between OD_600_ 0.4 and 0.8 were harvested in 50 mL Falcon tubes by centrifugation for 9 min at 3,220 × g, washed and resuspended at OD_600_ 0.4 to 0.8 in m-Sauton’s, then aliquoted into a 96-well black optical-bottom plate (Costar) in triplicate with drug or vehicle as appropriate. EtBr (Fisher Scientific, 10132863) in m-Sauton’s was added to each well to a final concentration of 1 μg/mL (25% of EtBr’s reported MIC for H37Rv) (101). Intrabacterial accumulation of EtBr was measured in situ using a plate reader (CLARIOstar, BMG LabTech) by fluorimetry at 530 ± 25 nm excitation and 590 ± 20 nm emission every 60 to 120 s. Measurements were continued until steady state was reached, typically after 90 min. For ease of comparison between drugs and experiments, the mean final 10 values measured in steady state under any one condition were expressed as a percentage of the mean final 10 values measured in steady state without drug present.

### Quantification of FITC-Rifampicin Efflux.

In contrast to the EtBr assay, the FITC-rifampicin assay requires intermittent sampling to measure supernatant fluorescence level. M-Sauton’s (as above) was used as a wash buffer throughout. Mid-log phase bacteria were harvested by centrifugation as above, resuspended in m-Sauton’s at OD_600_ 0.8 to 1.5, and then incubated for 10 min at 37 °C. To load the bacteria with FITC-rifampicin, FITC-rifampicin in DMSO was added to a final concentration of 2 μM and cells were incubated at 37 °C for 30 min. To remove free FITC-rifampicin, the cells were chilled for 10 min in an ice bath, washed three times in 50 mL buffer at 4 °C, and then resuspended on ice to OD_600_ 0.6 to 1.0. Drug or vehicle was added, and then the cells were transferred to a 37 °C water bath at the start of the time course. If required, an aliquot was then removed and heat-killed at 90 °C before being returned to 37 °C incubation. Supernatant fluorescence was analyzed at time points from bacterial suspensions by vortexing briefly, then passing a 300-μL aliquot through a 0.2 μm syringe filter (Pall Acrodisc) to remove bacteria. Alternatively, where large numbers of different drug treatments were tested simultaneously, 10 μL drug or vehicle was aliquoted at 100-fold the required concentration to wells of a 2-mL v-bottom deep well 96-well plate (Greiner Bio-one), and then 990 μL of bacterial suspension was added and mixed by multichannel. The plates were sealed with a Biorad adhesive micro-seal and incubated at 37 °C. At each time point, a single plate was centrifuged for 9 min at 3,220 × g, and then 200 μL of the supernatant was carefully removed without disturbing the pellet. FITC-rifampicin levels in each supernatant were quantified in triplicate by fluorimetry in black, optical bottom half-area 96-well plates (Greiner) using a plate reader (CLARIOstar, BMG LabTech) set to 483 ± 14 nm excitation and 530 ± 30 nm emission. For convenience, a standard 24-h final sampling time was adopted. For ease of comparison between drugs and experiments, the 24-h supernatant fluorescence value under any one condition was expressed as a percentage of the mean 24-h fluorescence value in the untreated group.

### Rifampicin and FITC-Rifampicin Efflux Competition.

Mid-log phase bacteria were harvested and prepared as above. FITC-rifampicin was added to a final concentration of 2 μM, and then the bacterial suspension was divided into two samples, which were treated either with 4 μM unlabeled rifampicin in DMSO, or DMSO alone. After incubation at 37 °C for 30 min, free FITC-rifampicin was removed and supernatant FITC-rifampicin levels were quantified as above.

### Measurement of Bacterial ATP Levels.

Mid-log phase bacteria were exposed to drug for 24 h at 37 °C and then 25 μL of bacteria was mixed with 25 μL of BacTiter Glo^TM^ reagent (Promega) as per manufacturer’s instructions and as previously described (102). Samples were mixed by orbital shaking at 100 rpm for 10 min at room temperature and then luminescence was measured in a black half-area 96-well plate using a plate reader (CLARIOstar, BMG LabTech) at 545 to 50 nm emission.

### MIC Assays.

We used a method adapted from Palomino et al. (103). A two-fold dilution series of drug at twofold the desired concentration was prepared in 100 μL volumes of 7H9 plus OADC without Tween-80 in a sterile flat-bottomed 96-well plate (Costar). A bacterial suspension at 10^5^ colony-forming units (CFU) per mL was prepared from a mid-logarithmic bacterial suspension in 7H9 plus OADC without Tween-80, then added to wells in 100 μL volumes. Each plate included growth control wells and drug-only negative controls. Parafilm-wrapped plates were incubated for 7 to 10 d until sufficient growth was observed in control wells by microscopy at 10× magnification using an AE2000 inverted microscope. Then 30 μL resazurin 0.02% + Tween-80 (7.7%) was added overnight. After 24 h, levels of the fluorescent product of resazurin, resorufin, were measured using a plate reader (CLARIOstar, BMG LabTech) at 530 ± 15 nm excitation and 590 ± 20 nm emission. The MIC was defined as the lowest concentration of drug preventing 95% of resorufin signal.

### Macrophage Growth and Infection.

THP-1 cells (ATCC) were cultured in Roswell Park Memorial Institute 1640 (RPMI) medium, supplemented with 10% fetal calf serum and 2 mM L-glutamine in a 37 °C incubator with 5% CO_2_. In each well of a sterile 24-well plate, 5 × 10^5^ cells per well were differentiated with 100 nM phorbol 12-myristate 13-acetate (PMA) for 48 h, washed once, then the media was replaced. After 24 h rest, the cells were infected at a multiplicity of infection (MOI) of one, incubated for 2 to 4 h, and then washed three times with PBS before replacing with fresh supplemented RPMI media containing 6 μg/mL streptomycin. For intramacrophage growth assays, streptomycin treatment was discontinued at the time of adding drug. At given time points, triplicate wells of infected cells were washed twice with PBS and incubated with 100 μL 0.1% Triton X-100 for 10 min before adding 900 μL PBS and scraping wells with a pipette tip. A serial dilution of 100 μL of each cell lysate was made in PBS and plated on 7H10 agar to enumerate CFU. Inhibitors were tested for toxicity to macrophages using exposure to resazurin as above with incubation for 4 to 6 h. For tolerance assays, triplicate wells were washed briefly with PBS and then with water. The cells were then incubated with 100 μL water per well at 37 °C for 15 min. Then, 900 μL 7H9 medium (supplemented with Middlebrook ADC and 0.05% Tween-80) was added, and the wells were scraped with a pipette tip. Lysates taken at 2 h or 96 h were then incubated for a further 48 h in the presence of drug. The lysates were plated for enumeration of CFU as described above, at the time of lysis and after 48 h further under drug exposure.

## Supplementary Material

Appendix 01 (PDF)

## Data Availability

All study data are included in the article and/or *SI Appendix*.
