# Supplementary material for: The human proton pump inhibitors inhibit Mycobacterium tuberculosis rifampicin efflux and macrophage-induced rifampicin tolerance
Source: Proc Natl Acad Sci U S A. 2023 Feb 10;120(7):e2215512120. doi: 10.1073/pnas.2215512120 (PMC7614234; doi:10.1073/pnas.2215512120)
Supplement: Supplementary file 1 — Appendix 01 (PDF) [file pnas.2215512120.sapp.pdf]

PNAS

Supporting Information for
The proton pump inhibitor class of drugs inhibits Mycobacterium tuberculosis rifampicin efflux, macrophage-induced rifampicin tolerance and intramacrophage bacterial growth

M. Alexandra Lake, Kristin N Adams, Feilin Nie, Elaine Fowler, Amit K Verma, Silvia Dei, Elisabetta Teodori, David R Sherman, Paul H Edelstein, David R Spring, Mark Troll, Lalita Ramakrishnan*

*lr404@cam.ac.uk

This PDF file includes:

Supp. Figures S1-8
Tables S1 to S4
Supplementary methods
Supp. Methods Figures A to H
SI References

Supporting Information

A B

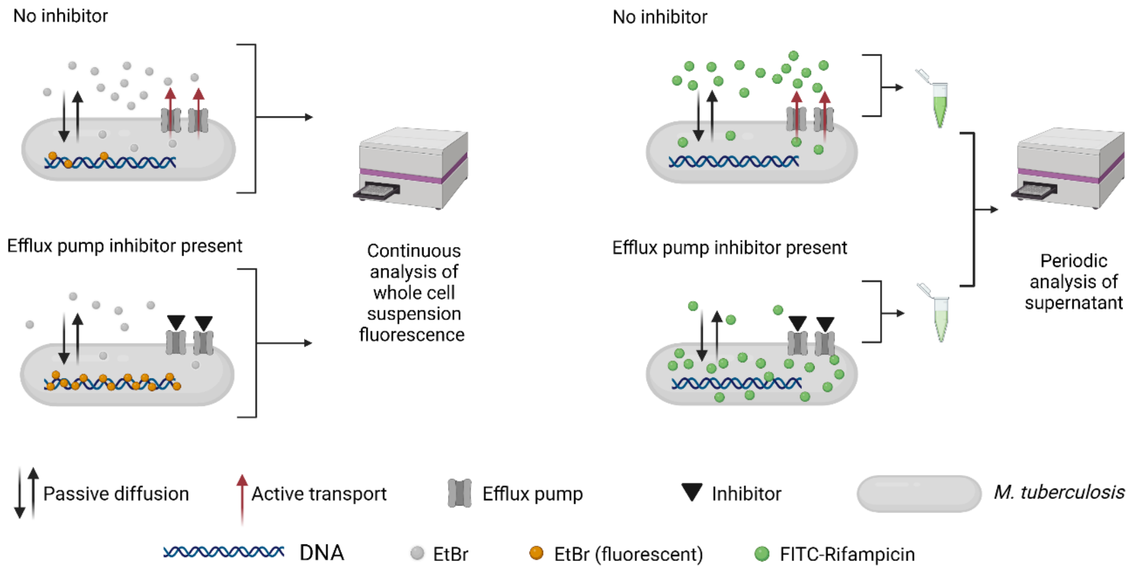

Fig. S1. Measurement of efflux activity using ethidium bromide (EtBr) or FITC-rifampicin. (**A**) Measurement of efflux activity using EtBr. Cells of Mtb are continuously immersed in a solution of EtBr. EtBr enters Mtb by passive diffusion. Its fluorescence increases when it intercalates with DNA inside the bacterial cell. EtBr can exit cells by passive diffusion or by active transport through efflux pumps. As entry into the cell become balanced by exit from the cell, intracellular EtBr levels reach a steady state, reflected by a steady fluorescence level. In the presence of an efflux pump inhibitor, the steady state intracellular EtBr level reached and resultant fluorescence level will be higher. (**B**) Measurement of efflux activity using FITC-rifampicin. Mtb is immersed in FITC-rifampicin for an initial incubation period at 37°C, then washed on ice to remove extracellular or adherent FITC-rifampicin. Cells are transferred to fresh media, and efflux begins when cells are warmed to 37°C. In contrast to the EtBr assay, which allows continuous, real-time measurement of fluorescence accumulation, the FITC-rifampicin assay requires intermittent sampling. At fixed time points, supernatants are separated from aliquots of the Mtb suspension by filtration or centrifugation. Fluorescence of the cell-free supernatant is then measured in a plate reader. In the presence of an efflux pump inhibitor, transport of intracellular FITC-rifampicin into the supernatant is reduced, resulting in lower supernatant fluorescence.

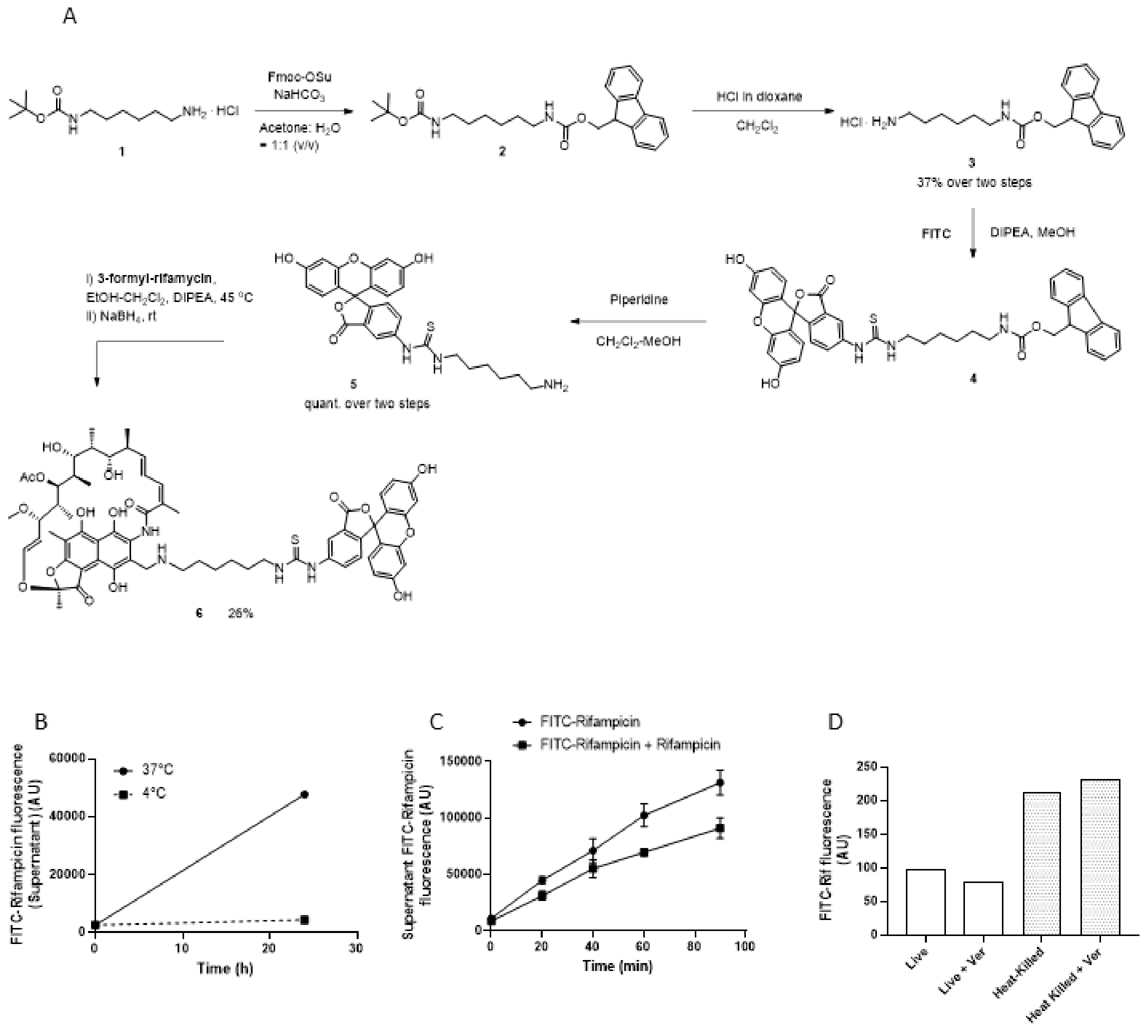

Figure S2*: Synthesis of FITC-rifampicin and assay validation.* (**A**) Schematic of FITC-rifampicin synthesis (**B**) FITC-rifampicin efflux is inhibited under cold conditions. Efflux of FITC-rifampicin from Mtb mc^{2}6206 at 37°C (solid line) and 4°C (dashed line). (**C**) Efflux of FITC-rifampicin from Mtb mc^{2}6206 into supernatant over time after loading with 2µM FITC-rifampicin alone, or co-loading with 2µM FITC-rifampicin and 4µM unlabelled rifampicin. Mean of two technical replicates; error bars show SEM. (**D**) Microbicidal treatment does not cause artefactual inhibition of efflux. Mtb was loaded with FITC-rifampicin, washed and either incubated at 37°C, or heat-killed at 90°C for 45 minutes then incubated at 37°C with or without 25µM verapamil (Ver). After 24 hours, supernatant FITC-rifampicin fluorescence was measured in each group.

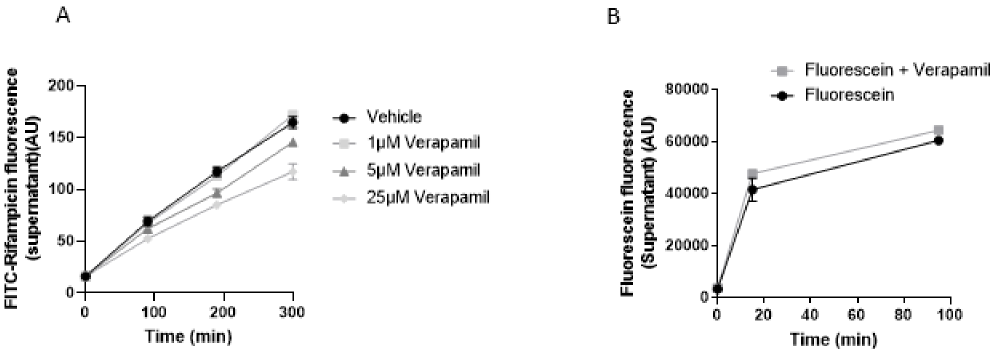

Figure S3: Verapamil inhibits Mtb efflux of FITC-rifampicin, but not unconjugated fluorescein. (**A**) Efflux of FITC-rifampicin into supernatant over time from Mtb $mc^2$6206 treated with Verapamil 0-25μM. Values represent mean of three technical replicates ± SEM. (**B**) Efflux of fluorescein into supernatant over time from Mtb $mc^2$6206 loaded with 4μM fluorescein and washed 3 times and resuspended in the usual way, in the presence or absence of 100μM verapamil.

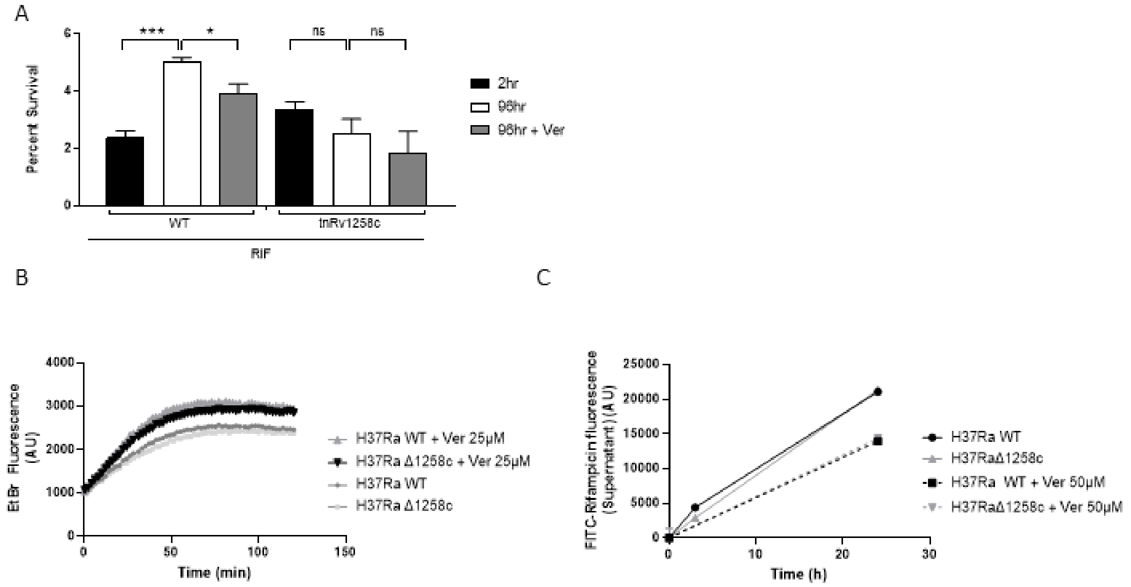

Figure S4: Loss of Rv1258c efflux activity of rifampicin is compensated for in broth culture but not macrophage culture. (**A**) Mtb CDC1551 lacking an intact Rv1258c gene cannot develop verapamil-sensitive macrophage-induced rifampicin tolerance. THP-1 macrophages were infected with Mtb CDC1551 parent strain or with Mtb CDC1551::Tn Rv1258c. Macrophages were lysed 2 hours (black bars) or 96 hours (white bars) after infection. The released bacteria were treated for an additional 48 hours with 1 μg/mL rifampicin with or without verapamil (Ver) before enumeration of CFU. Statistical analysis by ordinary one-way ANOVA with Dunnett's multiple comparisons test. Error bars represent SEM. *** $P < 0.005$, * $P < 0.5$. (**B,C**) Mtb H37Ra lacking an intact Rv1258c gene exhibits unaltered, verapamil-sensitive EtBr accumulation (**B**) and FITC-rifampicin efflux (**C**).

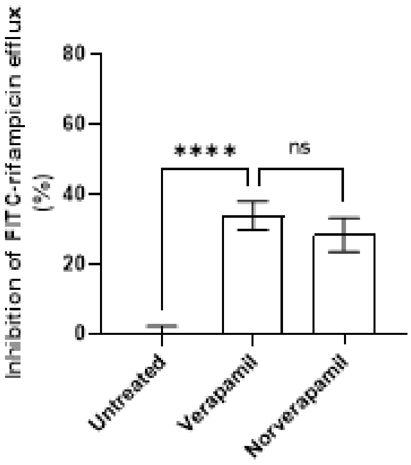

Figure S5: Norverapamil inhibits FITC-rifampicin efflux at the concentrations used to inhibit macrophage-induced tolerance in Mtb mc^{2}6206. Percent inhibition of FITC-rifampicin efflux from Mtb mc^{2}6206 due to verapamil 160µM or norverapamil 180µM, normalised to mean untreated value. Single experiment with three technical replicates. Error bars indicate mean ± SEM. Statistical analysis by one-way anova with Dunnett's multiple comparisons test. **** = P <0.0005

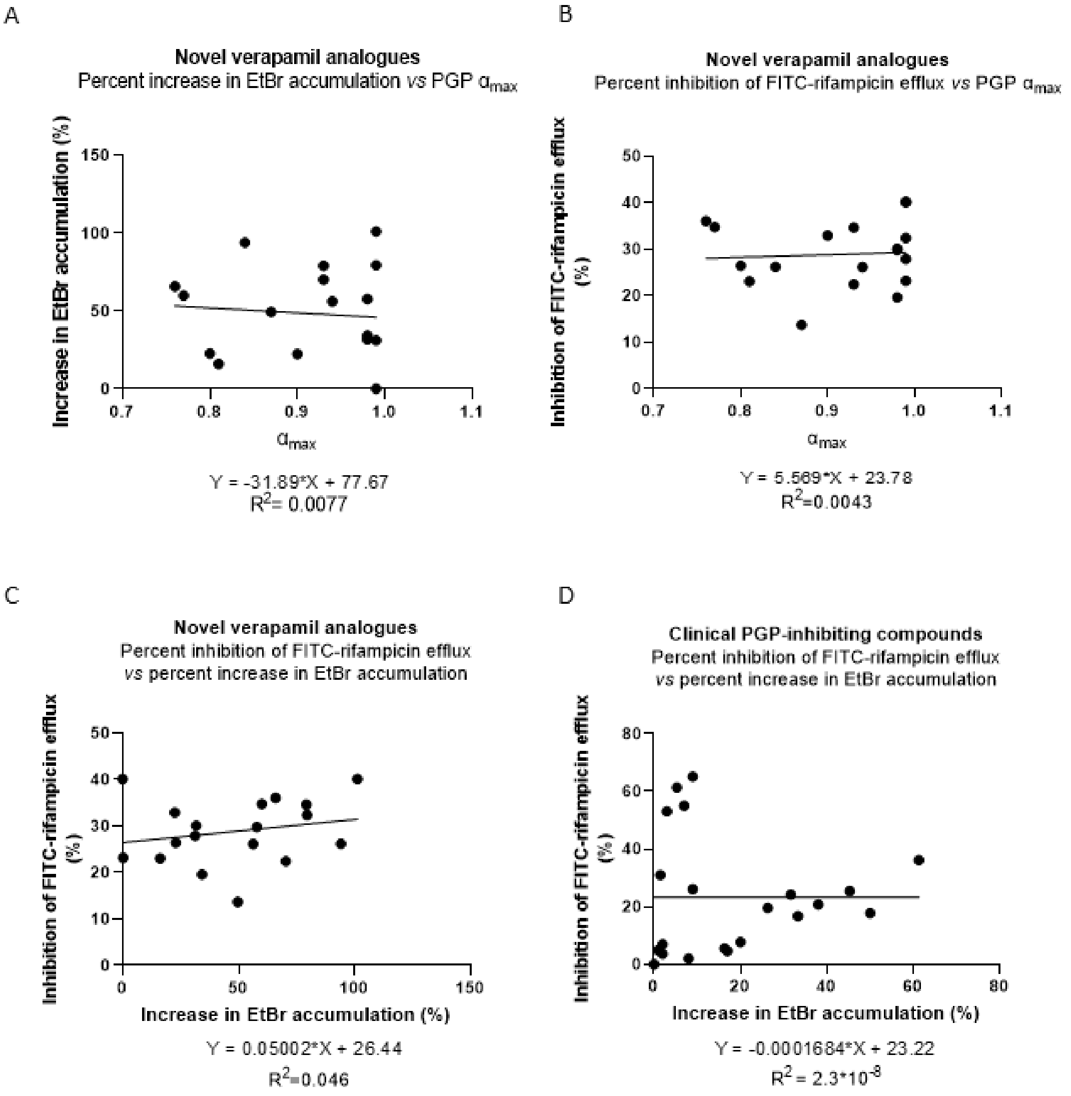

Figure S6: Increasing inhibition of Mtb efflux does not closely correlate with inhibition of PGP. Inhibition of FITC-rifampicin efflux does not closely correlate with inhibition of EtBr efflux. (**A-D**) Simple linear regression of mean value of 3 independent experiments. (**A**) Novel verapamil analogues: mean percent increase in EtBr accumulation versus PGP α_{max} per compound (**B**) Novel verapamil analogues: mean percent inhibition of FITC-rifampicin efflux versus PGP α_{max} per compound (**C**) Novel verapamil analogues: mean percent inhibition of FITC-rifampicin efflux against mean percent increase in EtBr accumulation. (**D**) Clinical PGP-inhibiting compounds: mean percent inhibition of FITC-rifampicin efflux against mean percent increase in EtBr accumulation per clinical drug listed in Fig. 3. PGP α_{max} represents the maximum increase in the nuclear concentration of the PGP substrate pirarubicin in eukaryotic pirarubicin-resistant cells that can be obtained with a given compound, where α varies between 0 (no inhibitor present) and 1 (when the amount of pirarubicin in resistant cells is the same as in sensitive cells); values as previously published (Dei *et al* 2019; Orlandi *et al* 2013; Dei *et al* 2001; Teodori et al 2005; Dei *et al* 2015). See Table S2 for detail.

A

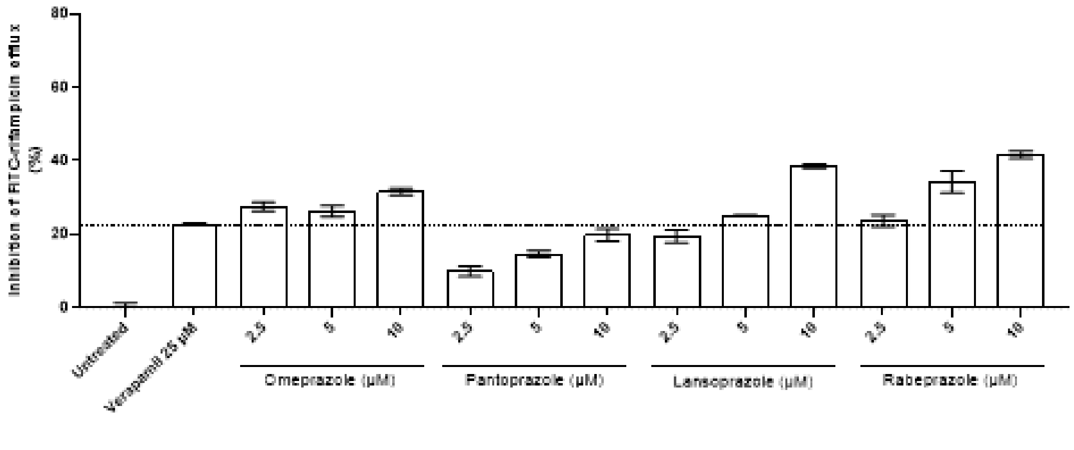

B

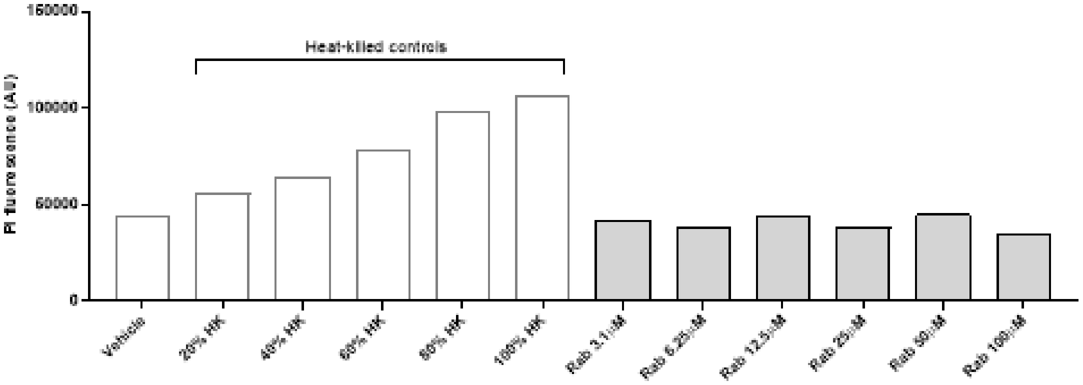

Figure S7: The proton pump inhibitors are effective inhibitors of FITC-rifampicin efflux below the screening concentration of 25µM. Exposure to rabeprazole does not alter bacterial cell permeability as measured by propidium iodide penetrance. (**A**) Percent inhibition of FITC-rifampicin efflux from Mtb mc^{2}6206 by the proton pump inhibitors, by comparison with verapamil at 25µM (dashed line). Values normalised to mean of untreated group. Representative of a single experiment with three technical replicates per group. Error bars represent SEM. All results were significantly different from the untreated group ($p<0.005$). Statistical analysis by one way anova with Dunnett multiple comparison test. (**B**) Propidium iodide (PI) fluorescence of Mtb mc^{2}6206, loaded with FITC-rifampicin and washed and resuspended in the usual way, then exposed to rabeprazole (Rab) for 24 hours at 37°C. At 24 hours, aliquots of heat killed (HK) cells were substituted for live vehicle-treated cells in known quantities as controls.

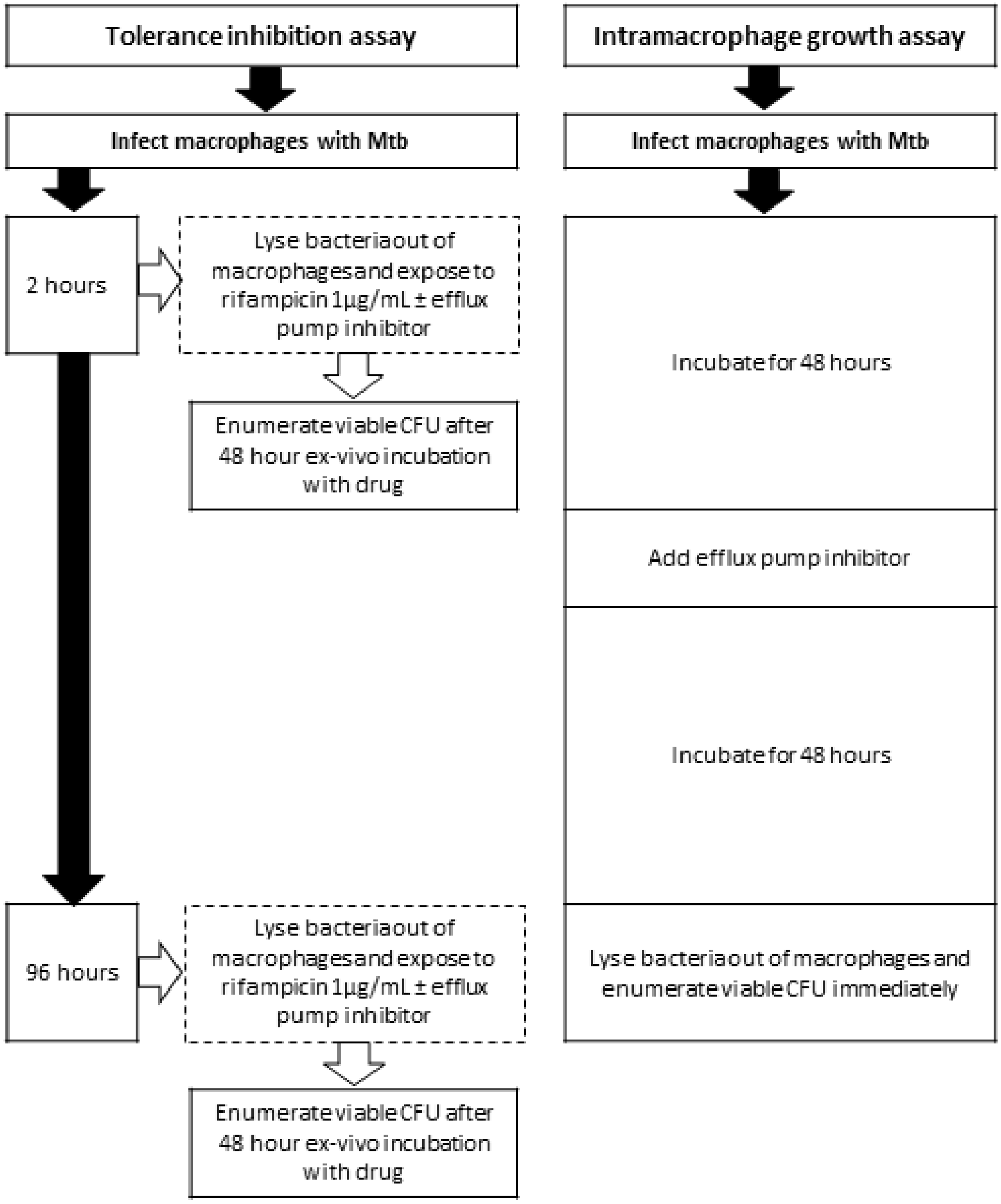

Figure S8: Schematic of experimental protocols for testing efficacy of novel efflux pump inhibitors on Mtb macrophage-induced rifampicin tolerance, or intramacrophage growth.

Table S1. MIC of key drugs in Mtb mc^{2}6206 and H37Rv. Representative of at least 2 independent experiments, except FITC-rifampicin, CCCP and lansoprazole in Mtb mc^{2}6206, which were tested once.

Drug	MIC (µM)<br>Mtb *mc^{2}6206*	MIC (µM)<br>Mtb *H37Rv*
**Rifampicin**	0.24	-
**FITC-rifampicin**	3.84	-
**Verapamil**	200	-
**CCCP**	25	-
**Bedaquiline**	0.125-0.25	-
**Rabeprazole**	100	200-400
**Lansoprazole**	100	800-1600
**Pantoprazole**	200	200-400
**Omeprazole**	400	400-1600

Table S2. Novel verapamil analogue terminology and compound structure

Compound	Molar mass / Key reference
**Compound 1 (ELF53.HCl)**	**Molar mass 626.13. Key reference (1)**
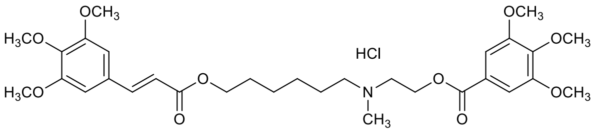	
**Compound 5 (RI4.HCl)**	**Molar mass 640.16. Key reference (1)**
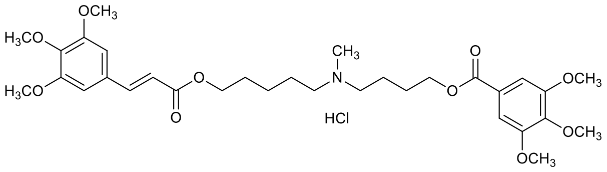	
**Compound 5D (MC260.HCl)**	**Molar mass 676.19. Key reference (2)**
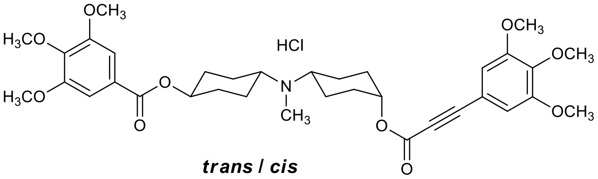	
**Compound 9 (ELF96.HCl)**	**Molar mass 640.16 Key reference (1)**
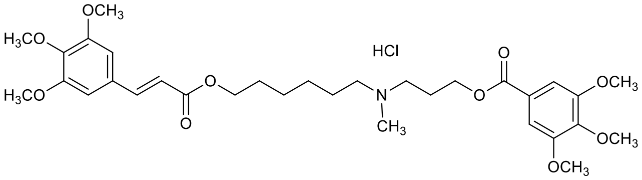	
**Compound 11 (ELF95.HCl)**	**Molar mass 650.2 Key reference (1)**
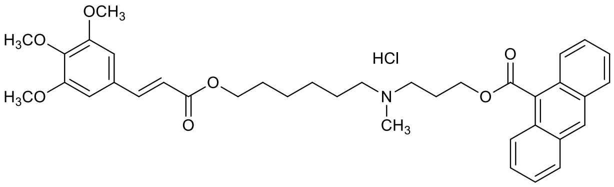	
**Compound 13 (ELF82.HCl)**	**Molar mass 640.16 Key reference (1)**
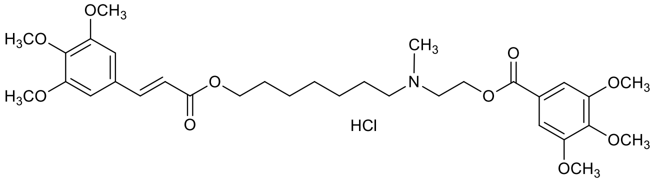	

Compound 15 (ELF83.HCl)

Molar mass 650.2 Key reference (1)

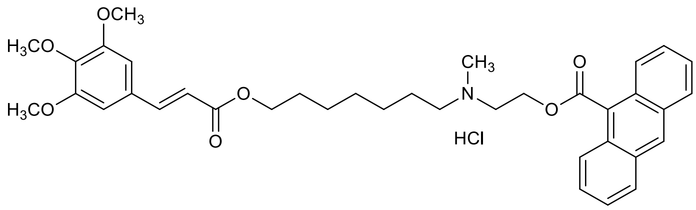

Compound 17-O (FC30.HCl)

Molar mass 534.13. Key reference (3), where named compound '17'.

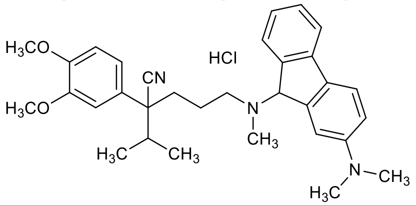

Compound 17-N (CF24.HCl)

Molar mass 654.19. Key reference (1)

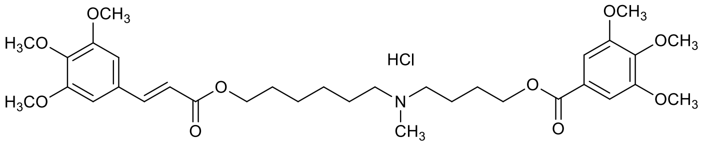

Compound 21 (ELF86.HCl)

Molar mass 654.19 Key reference (1)

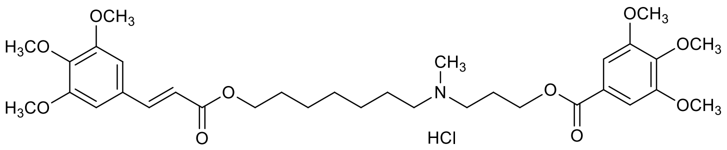

Compound 23 (ELF.HCl)

Molar mass 664.23. Key reference (1)

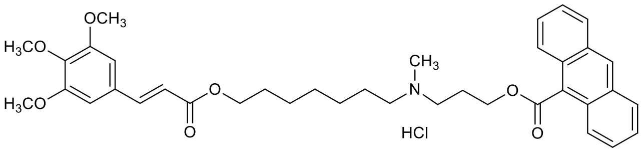

Compound 25 (CF34.HCl)

Molar mass 654.19. Key reference (1)

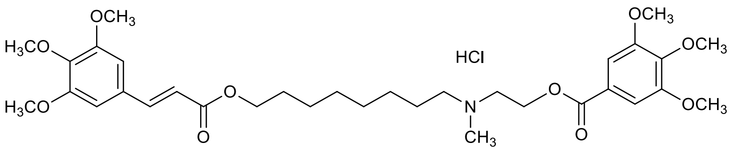

Compound V (ME30.HCl)

Molar mass 654.19. Key reference (1), where named 'Compound V' and (4), where named 'Compound 11'.

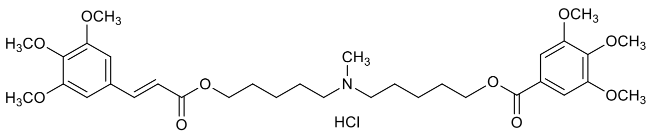

**Compound III (GDE6.HCl)** 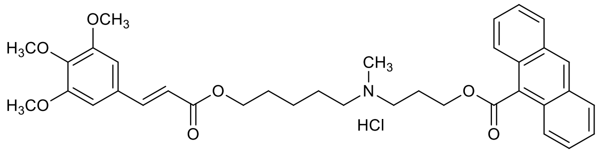	**Molar mass 636.17. Key reference (1), where named 'Compound III' and (5), where named 'Compound 16'.**
**Compound FC27 (FC27.oxa)** 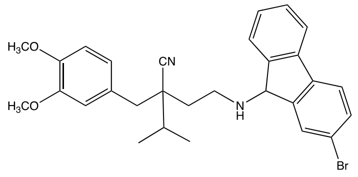	**Molar mass 560.64. Key reference (3), where named compound '8'.**
**Compound MC176 (MC176.HCl)** 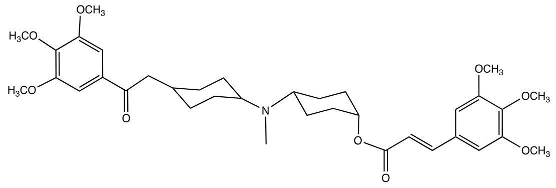	**Molar mass 678.21. Key reference (2), where named as 'Compound 1d'**
**Compound FRA58A (FRA58A.HCl)** 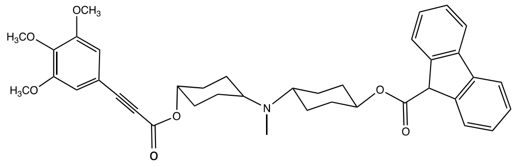	**Molar mass 736.29. Key reference (2), where named as 'Compound 6a'**

Table S3. Efficacy of novel verapamil analogs and clinical PGP inhibitor drugs against ethidium bromide (EtBr) accumulation. Data derived from main text Fig. 3 and 4. n = number experiments contributing to mean value. SEM, standard error of the mean.

Compound	Mean increase in EtBr accumulation (% ± SEM)	n
**Verapamil**	27.3 ± 7.3	6
**1**	60.0 ± 16.5	3
**5**	56.3 ± 11.1	3
**9**	22.6 ± 17.8	3
**11**	0.3 ± 0.3	3
**13**	101.2 ± 8.7	3
**15**	31.8 ± 9.3	3
**21**	31.3 ± 12.7	3
**23**	70.4 ± 13.8	3
**25**	79.2 ± 25.7	3
**17-N**	79.6 ± 17.2	3
**17-O**	16.2 ± 5.1	3
**5D**	0.0 ± 0.0	3
**FC27**	49.6 ± 18.6	3
**FRA58A**	66.0 ± 14.4	3
**III**	57.9 ± 13.8	3
**MC176**	34.3 ± 13.6	3
**V**	94.1 ± 19.0	3
**Atorvastatin**	50.0 ± 20.7	3
**Azelastine**	45.3 ± 16.9	3
**Caffeine**	2.0 ± 1.2	3
**Capsaicin**	16.3 ± 3.5	3
**Dexamethasone**	2.0 ± 0.6	3
**Ebastine**	33.3 ± 7.5	3
**Elacridar**	26.3 ± 5.8	3
**Ibuprofen**	1.0 ± 1.0	3
**Itraconazole**	-0.9 ± 2.6	3
**Lansoprazole**	5.3 ± 1.2	3
**Omeprazole**	7.0 ± 7.0	3
**Pantoprazole**	7.2 ± 4.6	3
**Piperine**	17.0 ± 5.3	3
**Rabeprazole**	9.0 ± 4.5	3
**Ritonavir**	38.0 ± 11.5	3
**Simvastatin**	20.0 ± 3.1	3
**Tariquidar**	9.0 ± 1.5	3
**Thioridazine**	61.3 ± 8.8	3
**Trimethoprim**	8.0 ± 0.6	3

Table S4. Efficacy of novel verapamil analogs and clinical PGP inhibitor drugs against FITC-rifampicin efflux. Data derived from main text Fig. 3 and 4. n = number experiments contributing to mean value. SEM, standard error of the mean.

Compound	Mean inhibition of FITC-rifampicin efflux (% ± SEM)	n
**Verapamil**	25.2 ± 2.4	7
**1**	34.8 ± 3.2	3
**5**	26.1 ± 4.0	3
**9**	32.9 ± 6.2	3
**11**	23.2 ± 4.9	2
**13**	40.1 ± 4.2	3
**15**	30.1 ± 1.1	3
**21**	27.9 ± 8.9	3
**23**	22.5 ± 0.2	2
**25**	34.7 ± 0.5	2
**17-N**	32.4 ± 7.0	3
**17-O**	23.1 ± 1.6	2
**5D**	40.1 ± 5.7	3
**FC27**	13.7 ± 0.5	2
**FRA58A**	36.1 ± 1.3	2
**III**	29.8 ± 7.2	3
**MC176**	19.6 ± 3.4	2
**V**	26.2 ± 8.8	3
**Atorvastatin**	11.1 ± 6.7	3
**Azelastine**	25.4 ± 1.3	3
**Caffeine**	3.6 ± 2.1	3
**Capsaicin**	5.5 ± 6.1	3
**Dexamethasone**	1.9 ± 5.5	3
**Ebastine**	16.7 ± 6.2	3
**Elacridar**	19.6 ± 9.0	4
**Ibuprofen**	3.1 ± 2.0	3
**Itraconazole**	31.0 ± 4.0	2
**Lansoprazole**	56.9 ± 4.8	3
**Omeprazole**	51.0 ± 4.9	3
**Pantoprazole**	43.0 ± 10.3	3
**Piperine**	4.8 ± 0.2	3
**Rabeprazole**	60.4 ± 4.9	3
**Ritonavir**	15.3 ± 7.3	3
**Simvastatin**	7.7 ± 4.3	3
**Tariquidar**	26.1 ± 4.3	4
**Thioridazine**	40.7 ± 4.7	3
**Trimethoprim**	0.1 ± 2.4	3

Supplementary Methods:

Synthesis of FITC-rifampicin

All reagents were obtained from commercial sources with no further purification. Dichloromethane and MeOH were distilled from calcium hydride. Reactions were under a dry nitrogen atmosphere. The synthetic route is summarized in Supp. Methods Fig. A. Preparative HPLC was carried out on an Agilent 1260 Infinity using a Supelcosil ABZ+PLUS column (250 mm x 21.2 mm, 5 µm) eluting with a linear gradient system (solvent A: 0.1% (v/v) TFA in water, solvent B: 0.05% (v/v) TFA in acetonitrile) over 20 min at a flow rate of 20 mL/min.
Proton (^{1}H) and carbon (^{13}C) nuclear magnetic resonance (NMR) spectra were recorded using a Bruker Ultrashield 400 spectrometer. Proton chemical shifts (δ) were measured in parts per million (ppm) relative to tetramethylsilane (δ =0 ppm), referenced to the appropriate solvent peak. Carbon chemical shifts (δ) were also measured in parts per million (ppm) relative to tetramethylsilane (δ =0), referenced to the appropriate solvent peak. Multiplicity is indicated using the following abbreviations: br, broad; s, singlet; d, doublet; t, triplet; q, quartet; quin, quintet; m, multiplet.
High resolution mass spectrometry (HRMS) was carried out using a Waters LCT Premier Time of Flight mass spectrometer or Micromass Quadrupole Time of Flight mass spectrometer. LCMS chromatographs were recorded on an Agilent 1200 series LC with an ESCi Multi-Mode ionisation waters ZQ spectrometer. The following conditions were used: solvent A: 10 mM ammonium acetate + 0.1% formic acid in water; solvent B: 95% acetonitrile + 5% H_2O + 0.05% formic acid; linear gradient: 0.0-0.7 mins: 0% B, 0.7-4.2 mins: 0-100% B, 4.2-7.7 mins: 100% B, 7.7-8.5 mins: 100-0% B; using a Supelcosil ABZ+PLUS column (33 mm × 4.6 mm,3 µm).*Tert*-butyl (6-aminohexyl)carbamate hydrochloride (Compound **1**, Supp. Methods Fig. A) (506 mg, 2.00 mmol, 1 eq) and sodium bicarbonate (504 mg, 6.00 mmol, 3 eq) were dissolved in acetone/H_2O (20 mL in total, 1:1, v/v) and Fmoc-OSu (675 mg, 2.00 mmol, 1 eq) was added. The mixture was stirred at room temperature overnight. The organic solvent was removed under reduced pressure and the residue partitioned between water and EtOAc. The organic phase was washed with 1N HCl, sat. aq. $NaHCO_3$ and brine, dried over $MgSO_4$, filtered and concentrated under reduced pressure.

A white solid **(2)** (Supp. Methods Fig. B) was formed and used in the next step without further purification. **2** was dissolved in CH_2Cl_2 (25 mL) and HCl in dioxane (4 M; 1 mL) was added. The reaction mixture was stirred for 5 hours at room temperature and white solids precipitated. The white solids were collected by filtration and washed with CH_2Cl_2 and Et_2O. After drying *in vacuo*, **3** (Supp. Methods Fig. C) (279 mg, 0.744 mmol, 37% yield over two steps) was obtained as a white solid.

^{1}H NMR (400 MHz, DMSO-d_6) δ = 7.88 (d, *J* = 7.6 Hz, 2H; H-15' and H-12'), 7.83 (d, *J* = 7.4 Hz, 1H; H-15), 7.67 (d, *J* = 7.4 Hz, 1H; H-12), 7.41 (td, *J* = 7.4, 1.1 Hz, 2H; H-14 and H-14'), 7.38-7.28 (m, 2H; H-13 and H-13'), 7.25 (t, *J* = 5.8 Hz, 0.5H; H-8 major), 6.64 (d, *J* = 6.5 Hz, 0.3H; H-8 minor), 6.27 (s, 1H; H-10), 4.29 (d, *J* = 6.8 Hz, 1H; H-9a), 4.19 (appt, *J* = 6.8 Hz, 1H; H-9b), 2.96 (appq, *J* = 6.6 Hz, 1H; H-7a), 2.94-2.85 (m, 1H; H-7b), 2.73 (t, *J* = 7.4 Hz, 2H; H-2), 1.55-1.45 (m, 2H; H-3), 1.38 (dt, *J* = 13.4, 6.7 Hz, 2H; H-6), 1.34-1.12 (m, 4H; H-4 and H-5); **^{13}C NMR** (100 MHz, DMSO-d_6) δ = 144.3 (C-11'), 143.0 (C-17), 141.2 (C-16), 139.8 (C-16'), 137.9 (C-11), 129.4 (C-14'), 128.1 (C-14), 127.8 (C-13), 127.5 (C-13'), 125.6 (C-12), 121.8 (C-12'), 120.6 (C-15'), 120.5 (C-15), 110.2 (C-10), 29.6 (C-6), 27.8 (C-3), 26.0 (C-4 and C-5); **LCMS** (ESI+) *m/z* = 339.3 $[M+H]^+$ **(**Supp. Methods Fig. G).

3 (37 mg, 0.10 mmol, 1 eq) was dissolved in MeOH (2 mL) and DIPEA (70 µL, 0.40 mmol, 4 eq) was added. FITC (39 mg, 0.10 mmol, 1 eq) dissolved in MeOH (1 mL) was added to the mixture. The mixture was stirred at room temperature for 4 hours. LCMS indicated completed turnover. The reaction solvent was removed under a stream of nitrogen. The red residue **(4)** (Supp. Methods Fig. D) was used in the next step without further purification. **LCMS** (ESI) *m/z* = 728.2 $[M+H]^+$, 726.3 $[M-H]^-$.

4 (crude) was dissolved in MeOH (0.5 mL) and 20% piperidine in CH_2Cl_2 (3 mL in total) added. The mixture was stirred at room temperature for 2.5 hours. LCMS indicated completed turnover.

The reaction solvent was removed under a stream of nitrogen. The residue was re-dissolved in water. White solid formed and was filtered off. The filtrate was purified using preparative HPLC (10-60% B). After lyophilisation of HPLC fractions, **5** (Supp. Methods Fig. E) (51 mg, 0.10 mmol, quant. over two steps) was obtained as a yellow powder. **HPLC** t_r = 5.81 min (30-100% B), peak area 96%; **LCMS** (ESI) *m/z* = 504.3 $[M\text{-}H]^-$; **HRMS** (ESI+) *m/z* = 506.1727 $[M+H]^+$ found, $C_{27}H_{28}O_5N_3{}^{32}S_1{}^+$ required 506.1744.

3-Formyl rifamycin (36 mg, 0.050 mmol, 1 eq) and **5** (25 mg, 0.050 mmol, 1 eq) were dissolved in a mixture of EtOH and CH_2Cl_2 (4 mL in total, 1:1, v/v). DIPEA (1 drop) and 4 Å molecular sieves were added. The mixture was heated at 45°C overnight. LCMS indicated incomplete turnover. However, longer reaction time led to the decrease of product; therefore, the reaction was stopped. Solvent was reduced to half volume. $NaBH_4$ (0.95 mg, 0.025 mmol, 0.5 eq) was added followed by a few drops of water. The reaction solvent was removed under a stream of nitrogen. The residue was purified using preparative HPLC (40-70% B). After lyophilisation of HPLC fractions, **6** (Supp. Methods Fig. F, NMR spectra Supp. Methods Figure H) (16 mg, 0.013 mmol, 26%) was obtained as a yellow powder.

HPLC t_r = 12.38 min (5-100% B), peak area 94%; **^{1}H NMR** (500 MHz, DMSO-d_6) spectrum was given below, but full assignment was not conducted due to the rotamers at room temperature and decomposition at elevated temperature NMR; **LCMS** (ESI) *m/z* = 608.3 $[M+2H]^{2+}$, 1215.4 $[M+H]^+$, 1213.2 $[M\text{-}H]^-$; **HRMS** (ESI+) *m/z* = 1215.4834 $[M+H]^+$ found, $C_{65}H_{75}O_{17}N_4{}^{32}S_1{}^+$ required 1215.4842.

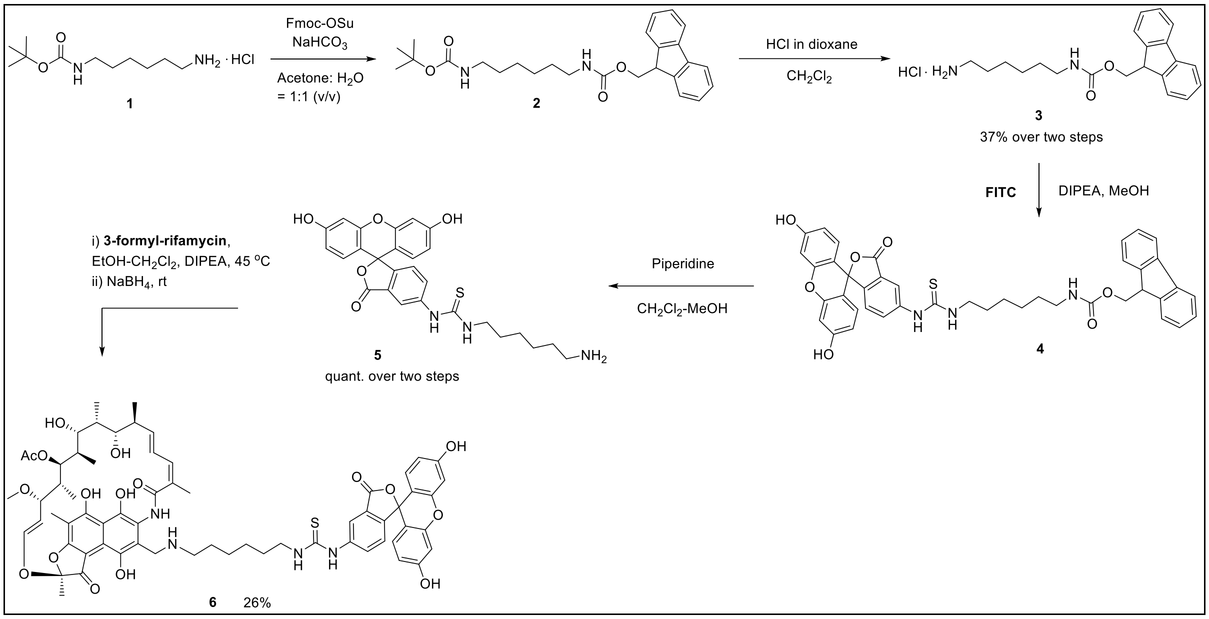

Supp. Methods Fig. A. FITC-rifampicin synthetic route.

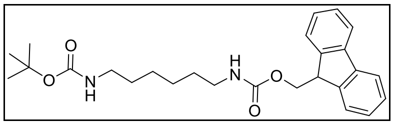

Supp. Methods Fig. B. (9H-Fluoren-9-yl)methyl tert-butyl hexane-1,6-diyldicarbamate (**2**)

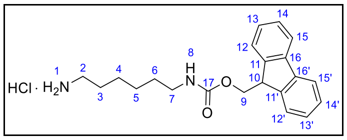

Supp. Methods Fig. C. (9H-Fluoren-9-yl)methyl (6-aminohexyl)carbamate hydrochloride (**3**)

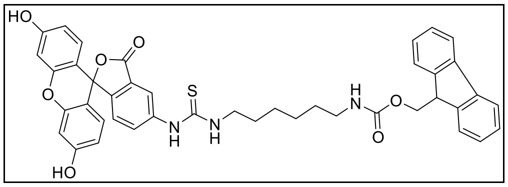

Supp. Methods Fig. D. (9H-Fluoren-9-yl)methyl (6-(3-(3',6'-dihydroxy-3-oxo-3H-spiro[isobenzofuran-1,9'-xanthen]-5-yl)thioureido)hexyl)carbamate (**4**)

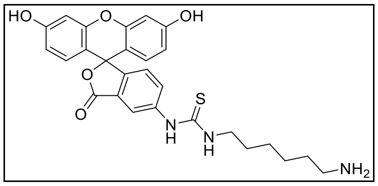

Supp. Methods Fig. E 1-(6-Aminohexyl)-3-(3',6'-dihydroxy-3-oxo-3H-spiro[isobenzofuran-1,9'-xanthen]-5-yl)thiourea (**5**)

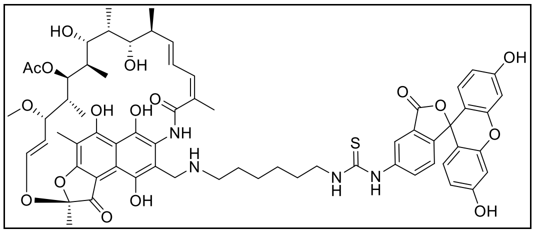

Supp. Methods Fig. F. FITC-rifampicin (**6**)

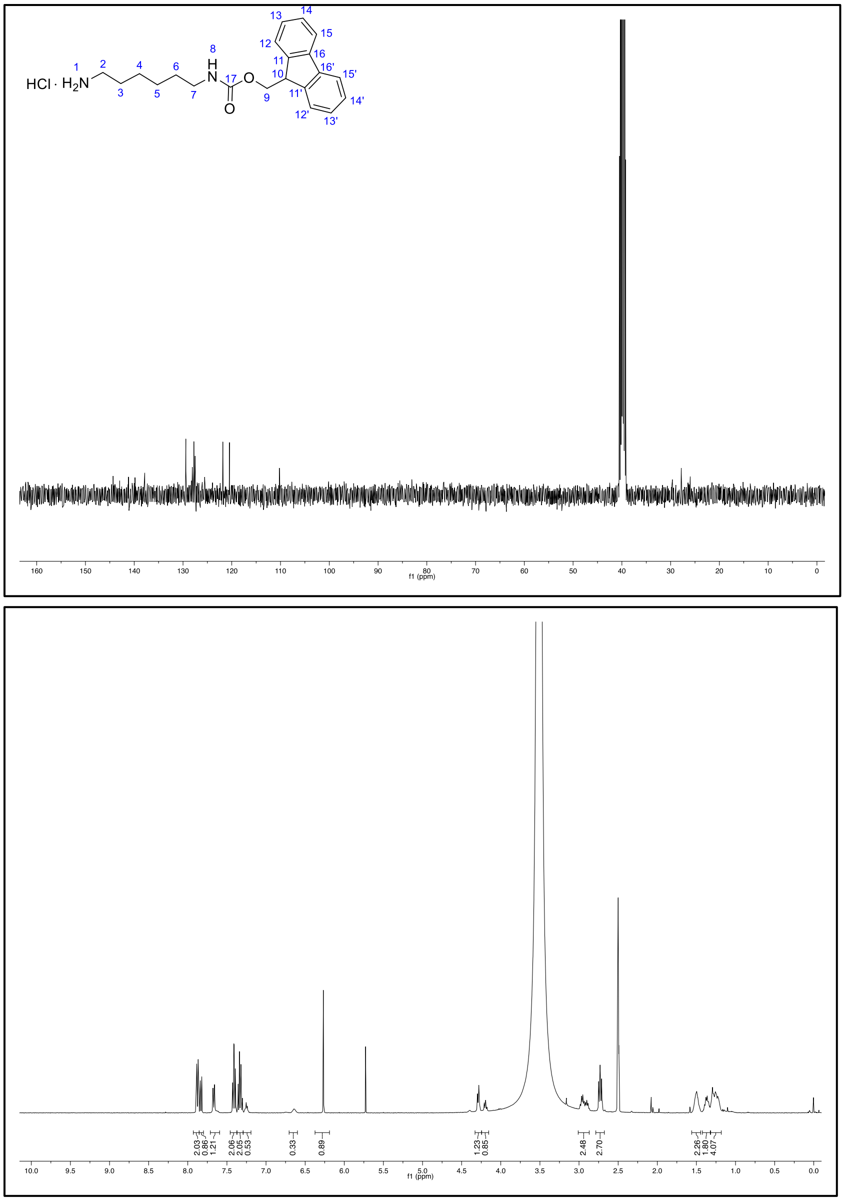

Supp. Methods Fig. G. NMR spectra **3**

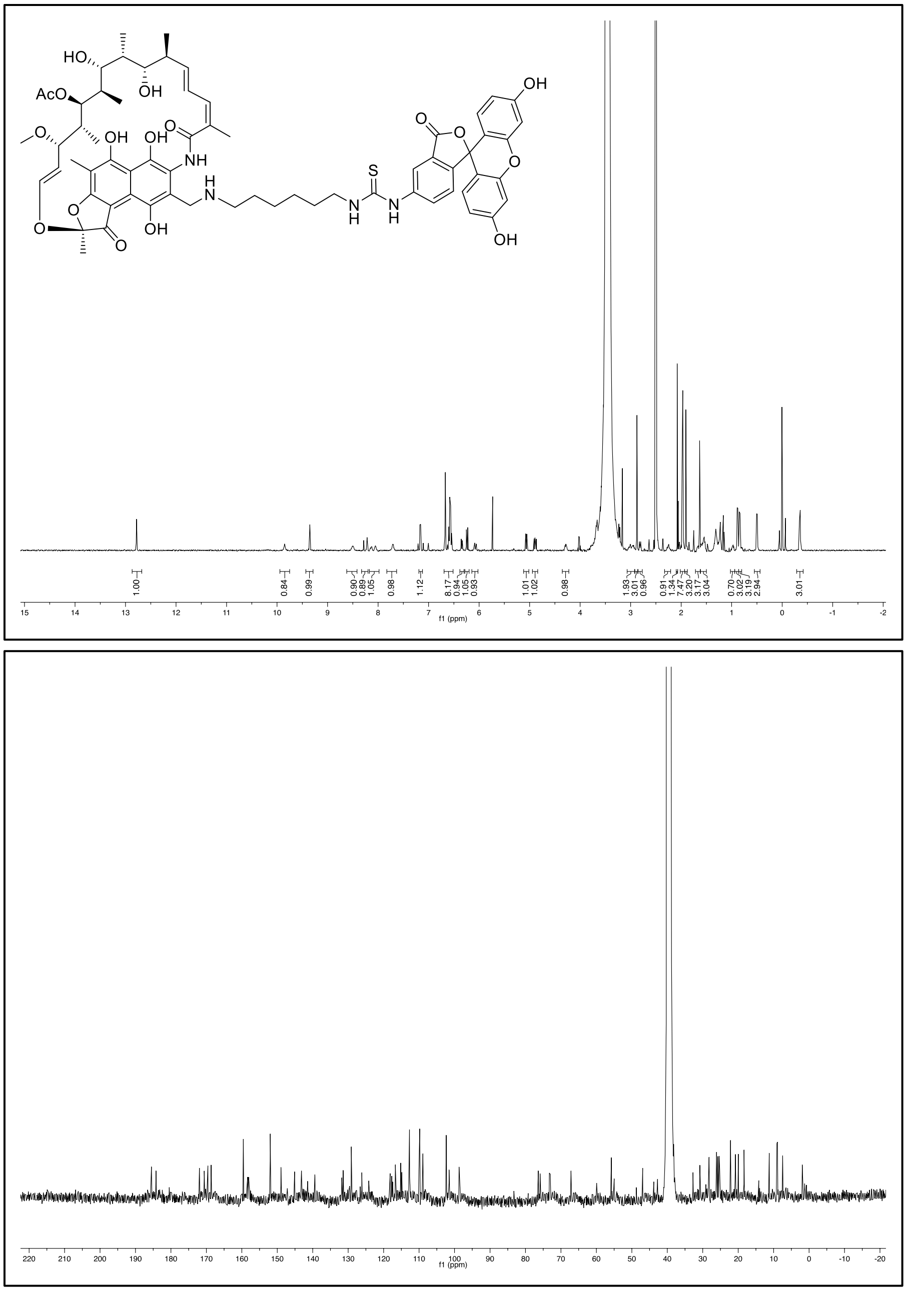

Supp. Methods Figure H. NMR spectra **6**

SI References

1. S. Dei, *et al.*, Modulation of the spacer in N,N-bis(alkanol)amine aryl ester heterodimers led to the discovery of a series of highly potent P-glycoprotein-based multidrug resistance (MDR) modulators. *Eur. J. Med. Chem.* **172**, 71–94 (2019).
2. F. Orlandi, *et al.*, New structure-activity relationship studies in a series of N,N-bis(cyclohexanol)amine aryl esters as potent reversers of P-glycoprotein-mediated multidrug resistance (MDR). *Bioorganic Med. Chem.* **21**, 456–465 (2013).
3. S. Dei, *et al.*, Structure-activity relationships and optimisation of the selective MDR modulator 2-(3,4-dimethoxyphenyl)-5-(9-fluorenylamino)-2-(methylethyl) pentanenitrile and its N-methyl derivative. *Bioorganic Med. Chem.* **9**, 2673–2682 (2001).
4. E. Teodori, *et al.*, Exploratory chemistry toward the identification of a new class of multidrug resistance reverters inspired by pervilleine and verapamil models. *J. Med. Chem.* **48**, 7426–7436 (2005).
5. S. Dei, *et al.*, Multidrug resistance (MDR) reversers: High activity and efficacy in a series of asymmetrical N, N-bis(alkanol)amine aryl esters. *Eur. J. Med. Chem.* **87**, 398–412 (2014).
